# Genetic epidemiology and plasmid-mediated transmission of *mcr-1* by *Escherichia coli* ST155 from wastewater of long-term care facilities

**DOI:** 10.1128/spectrum.03707-23

**Published:** 2024-02-14

**Authors:** Jun Feng, Miao Pan, Yuan Zhuang, Jiayuan Luo, Yong Chen, Yitong Wu, Jiayi Fei, Yanqi Zhu, Zhen Xu, Zhengan Yuan, Min Chen

**Affiliations:** 1 Shanghai Municipal Center for Diseases Control and Prevention, Shanghai, China; Fujian Agriculture and Forestry University, Fuzhou, China

**Keywords:** wastewater, long-term-care facility, *mcr-1*, plasmid, *Escherichia coli* ST155

## Abstract

Long-term care facilities (LTCFs) for older people play an important and unique role in multidrug-resistant organism transmission. Herein, we investigated the genetic characteristics of mobile colistin resistance gene (*mcr-1*)-carrying *Escherichia coli* strains isolated from wastewater of LTCFs in Shanghai. Antimicrobial susceptibility test was carried out by agar dilution methods. Whole-genome sequencing and plasmid sequencing were conducted, and resistance genes and sequence types of colistin in *E. coli* isolates were analyzed. Core genome multilocus sequence typing (cgMLST) analysis was performed by the Ridom SeqSphere+ software. Phylogenetic tree through the maximum likelihood method was constructed by MEGA X. Out of 306 isolates, only 1 *E. coli* named ECSJ33 was found, and the plasmid pECSJ33 from ECSJ33 harbored the *mcr-1* gene that was located with 59,080 bp belonging to IncI2 type. The plasmid pECSJ33 was capable of conjugation with an efficiency of 2.9 × 10^−2^. Bioinformatic analysis indicated pECSJ33 shared backbone with the previously reported *mcr-1*-harboring pHNGDF93 isolated from fish source. Moreover, the cgMLST analysis revealed that ECSJ33 belongs to different lineages from those reported from previous *E. coli* strains but shared high similarity to NCTC11129 in cluster 11. The phylogenetic tree revealed MCR-1 of ECSJ33 in this study was mostly of animal food origin and that they were closely related. Our study firstly reports detection of genome sequence of a multidrug-resistant *mcr-1*-harboring *E. coli* ST155 from wastewater of LTCF source in China. The data may prove that the plasmid pECSJ33 belongs to food origin and help to understand the antimicrobial resistance mechanisms and genomic features of colistin resistance under One Health approach.

**IMPORTANCE:**

One *Escherichia coli* named ECSJ33 was found from wastewater of a long-term care facility (LTCF) and the plasmid pECSJ33 from ECSJ33 harbored the mobile colistin resistance gene (*mcr-1*) that was located with 59,080 bp belonging to IncI2 type, which was capable of conjugation with an efficiency of 2.9 × 10^-2^. This paper firstly reports an *mcr-1*-carrying *E. coli* strain ST155 isolated from LTCF in China. Comparative genomics analysis indicated pECSJ33 shared backbone with the previously reported *mcr-1*-harboring pHNGDF93 isolated from fish source. The phylogenetic tree revealed MCR-1 protein of ECSJ33 in this study was mostly of animal food origin and that they were closely related. Therefore, the pECSJ33 could be considered as food-origin transmission *mcr-1*-harboring plasmid.

## INTRODUCTION

*Enterobacteriaceae* isolates, which carry plasmid-borne carbapenemase and extended-spectrum β-lactamase (ESBL) genes (1), could generate resistance to multiple drugs and contribute to the spread of multidrug-resistant (MDR) bacteria in human populations. The frequent usage of β-lactams and carbapenems over the past several decades has led to the increased use of colistin, which is considered the last therapeutic option for treating infections caused by such organisms (2, 3). Currently, the efficacy of colistin has been challenged by the emergence of plasmid-mediated mobile colistin resistance gene (*mcr-1*), which was found in *Enterobacteriaceae* in 2016 (4), and has since been disseminated in animals, meat products, humans (both fecal carriage and infections), and the environment in over 50 countries, covering six continents (5–8). In China, colistin has been adopted for treating carbapenem-resistant Enterobacterales (CRE) infections since 2018 (9), thereby increasing the potential risk of the dissemination of *mcr-1*. Therefore, to effectively control *mcr-1* gene prevalence from human, animal, and environment sources is crucial.

The spread of colistin resistance is driven by a number of factors, including the overuse and misuse of colistin, the lack of new antibiotics in development, and the dissemination of colistin resistance genes through horizontal gene transfer (10–12). Hospital wastewater is known to be a reservoir for antibiotic-resistant bacteria and resistance genes, and its contamination with *mcr-1*-harboring bacteria is a serious public health concern (13, 14). Furthermore, residents of long-term care facilities (LTCFs) are recognized as being at increased risk of colonization/infection with MDR organisms because of age-associated morbidities, exposure to recurrent antibiotic courses, and frequent referral to and from acute care hospitals (15). LTCFs were reported as the carriers of CRE, most were ESBL and *Klebsiella pneumoniae* (16–18). The discharge of wastewater into the environment can facilitate the spread of these bacteria and genes. Therefore, the detection and characterization of antibiotic-resistant bacteria in LTCF wastewater are crucial for understanding the spread of resistance genes and developing effective strategies for their control. In recent years, there has been growing awareness of antimicrobial resistance pollution in aquatic environments, leading to increased research on the presence and coexistence of antibiotics, and antimicrobial resistance genes (ARGs) (19, 20).

Shanghai is one of the most populous and economically important cities in China, with a large concentration of industrial and commercial activities. Wastewater treatment plants (WWTPs) in Shanghai receive effluent from various sources, including hospitals, food-processing industries, and residential areas, making them potential hotspots for the dissemination of antimicrobial resistance. Several studies have reported the presence of *mcr-1* in *Escherichia coli* isolates from wastewater in global and other regions of China (21–24); however, there is limited information on the prevalence and genetic diversity of *mcr-1* in *E. coli* isolated from LTCF wastewater in Shanghai.

In this study, we aimed to investigate the prevalence, genetic characteristics, and antimicrobial susceptibility profiles of *mcr-1*-harboring *E. coli* isolated from LTCF wastewater in Shanghai, China. We also aimed to assess the potential risk of transmission of *mcr-1*-harboring *E. coli* from wastewater to humans and the environment. Our findings will provide valuable insights into the epidemiology and ecology of *mcr-1*-harboring *E. coli* in wastewater and the potential implications for public health.

## MATERIALS AND METHODS

### Sample collection and strain isolation

A total of 306 medical institutions as sentinel surveillance port were randomly selected in 15 districts in Shanghai during May to August 2022. The sampling point is set at the sewage outlet of the medical institution, and the sampling point management file shall be established, including the sampling name, Global Position System and number of the sampling point, and the sampling frequency and pollution factors. The sampling container is a 500 mL sterilized sampling bottle, which is rinsed three times with a water sample before sampling, followed by direct sampling at the sampling depth. Water samples (50 mL each) were collected aseptically and kept in 4°C during transportation and sent to the laboratory within 4 h of collection. After sedimentation by gravity, the supernatants were filtered using membrane filters and subsequently placed into *Enterobacteriaceae* enrichment broth (Trypticase Soy Broth, COMAGAL Microbial Technology, Shanghai, China) for overnight enrichment. Next, 200 µL of bacterial solutions was inoculated into MacConkey agar medium (COMAGAL Microbial Technology, Shanghai, China) containing 2 µg/mL colistin overnight at 37°C. The single colonies were then identified by matrix-assisted laser desorption-ionization time of flight mass spectrometry using the VITEK MS system (BioMérieux Shanghai Co. Limited). The score cut-off of ≥2.000 was applied for species-level identification according to the manufacturer’s recommendation.

### Detection of colistin-resistant *E. coli* and screening of *mcr-1* to *mcr-10* gene

The positive red colonies were further confirmed and selected by amplifying the *mcr-1* to *mcr-10* gene via real-time PCR (RT-PCR). Basic clinical data, including gender, age, and date of isolation, were collected for patients from whom the *mcr-1*-harboring strains were isolated. The genomic DNA from each of the strains was extracted by boiling and freeze-thawing processes, and the resulting supernatant was used as the template. The specific primer for *mcr-1* used in this study was reported previously (25). The primers of *mcr*-2 to *mcr*-5 were derived as reported by Rebelo et al. (26). As for *mcr-*6 to *mcr*-10, the primers were derived from Maria et al. (27).

### Antimicrobial susceptibility testing

This testing was performed by the broth microdilution method using 28 antimicrobial agents (Shanghai Fosun Biological Technology Co., Ltd., China) including ampicillin (AMP), ampicillin/sulbactam 2:1 ratio (AMS), tetracycline (TET), chloramphenicol (CHL), trimethoprim/sulfamethoxazole (SXT), cefazolin (CFZ), cefotaxime (CTX), ceftazidime (CAZ), cefoxitin (CFX), gentamicin (GEN), imipenem (IMP), nalidixic acid (NAL), azithromycin (AZI), tigecycline (TIG), ciprofloxacin (CIP), amoxicillin/clavulanic acid (AMC), cefotaxime/clavulanic acid (CTC), ceftazidime/clavulanic acid (CAC), colistin (CT), aztreonam (ATM), cefuroxime (CXM), amikacin (AMI), cefepime (CPM), meropenem (MEM), levofloxacin (LEV), ertapenem (ETP), ceftazidime/avibactam (CZA), streptomycin (STR), and norfloxacin (NOR). *E. coli* ATCC 25922 strain was used as quality control. The minimum inhibitory concentrations (MICs) of colistin were determined and interpreted using the European Committee on Antimicrobial Susceptibility Testing (EUCAST) guidelines with 2 µg/mL for colistin resistance (https://view.officeapps.live.com/op/view.aspx?src=https%3A%2F%2Fwww.eucast.org%2Ffileadmin%2Fsrc%2Fmedia%2FPDFs%2FEUCAST_files%2FBreakpoint_tables%2Fv_13.0_Breakpoint_Tables.xlsx&wdOrigin = BROWSELINK). Strains resistant to three or more classes of antimicrobial agents were defined as multidrug-resistant bacteria.

### Conjugation assay

To investigate whether the *mcr-1* gene was present on a transferable plasmid, we performed plasmid conjugation transfer experiments using *E. coli* C600 (rifampicin resistant) as the recipient. The donor and recipient bacterium were cultured in Luria-Bertani (LB) liquid medium at 37°C until optical densiy of 600 nm (OD_600_) reached 0.5, and then, the donor/recipient bacterium were mixed at a ratio of 1:3. The mixture was added into 5 mL LB liquid medium incubated for mating for 16–18 h at 37°C. The transconjugants were selected on MacConkey agar containing 40 µg/mL rifampicin and 2 µg/mL colistin. Putative transconjugant was confirmed by antimicrobial susceptibility testing and RT-PCR, and the transfer frequency was calculated by transconjugants/donors as previously described (28).

### Whole genome sequencing (WGS) analysis

Genomic DNA was extracted using the QIAGEN genomic DNA purification kit (QIAGEN, Germany). Sequencing libraries were generated using the TruSeq DNA Sample Preparation Kit (Illumina, USA). The genome sequencing was then performed with standard protocol and were sequenced with 150 bp paired-end strategy by using the Illumina Novaseq 6000 (Sangon Biotech Company, Shanghai, China). Bacterial genome assembly was proceeding after adapter contamination removal and data filtering by using SPAdes software (version 3.12.3) (29) and A5-miseq to constructed scaffolds and contigs. Function annotation was completed by BLAST search against different databases and were annotated using PATRIC (version 3.6.9).

The assembled contigs were subsequently queried with PubMLST (https://pubmlst.org/), PlasmidFinder 2.1 (https://cge.cbs.dtu.dk/services/PlasmidFinder/), CARD (The Comprehensive Antibiotic Resistance, https://card.mcmaster.ca/), and VFDB (Virulence Factors of Pathogenic Bacteria, http://www.mgc.ac.cn/VFs/main.htm) available from the Center for Genomic Epidemiology (http://www.genomicepidemiology.org) for multilocus sequence typing (MLST), plasmid replicon typing, antimicrobial resistance gene identification, and virulence gene identification, respectively. The bacterial species differences and genome-wide similarities were generated by FastANI (Fast Average Nucleotide Identity, version 1.33).

### Plasmid sequencing

Plasmid DNA was extracted from *E. coli* C600 transformants harboring the *mcr-1* plasmid with the QIAGEN plasmid miniprep kit (QIAGEN, Germany). Plasmid DNA was excised from the gel and purified using the Wizard SV gel and PCR clean-up system (Promega), which was used to prepare sequencing libraries using the Nextera XT DNA sample preparation kit (Illumina, USA). Next-generation sequencing analysis of 150 bp paired-end sequencing was performed on the MiSeq platform (Illumina, USA). The contigs obtained after *de novo* assembly as described above were subjected to gap-closing PCR and Sanger sequencing to determine the complete nucleotide sequence. The obtained sequence data were submitted to the DFAST for annotation. The linear comparison of complete plasmid sequences was created by Easyfig (http://mjsull.github.io/Easyfig/). The plasmid construction map was generated by SnapGene 6.1.2 software (Insightful Science, USA).

### Phylogenetic analysis

The complete genomes of *mcr-1*-positive *E. coli* (MCRPEC) were used for phylogenetic analysis. The identification of core genes and core genome was performed using Roary, and then the core genomic alignment was used to construct a maximum likelihood phylogeny on MEGA X (Mega Limited, Auckland, New Zealand). The core genome MLST type (cgMLST) analysis was performed by the Ridom SeqSphere+ software (GmbH, Münster, Germany). In *E. coli*, a cgMLST cluster was defined if having ≤10 non-identical target gene (30). Consequently, 3,179 core genes were used to construct a minimum spanning tree and look for allelic differences.

For MCR-1 protein phylogenetic analysis, the MCR-1 and MCR-1-like proteins’ homologous sequences were extracted from the NCBI through BLASTp search (https://blast.ncbi.nlm.nih.gov/Blast.cgi, accessed on 30 September 2023), with MCR-1 protein of ECSJ33 in this study obtained from the sequencing data. Aligned sequences of MCR-1 obtained from ClustalW version 2.0 (http://www.clustal.org/) were used to construct a phylogenetic tree through the maximum likelihood method of MEGA X. To confirm the results, 1,000 bootstrap repetitions were used.

### Statistical analysis

Kruskal-Wallis rank sum test was used for statistical analysis, and *P* < 0.05 was considered as statistically significant. All statistical tests were carried out by SPSS v26.0 (IBM, USA).

## RESULTS

### *mcr-1*-harboring *E. coli* isolate detection

**Fig 1:**
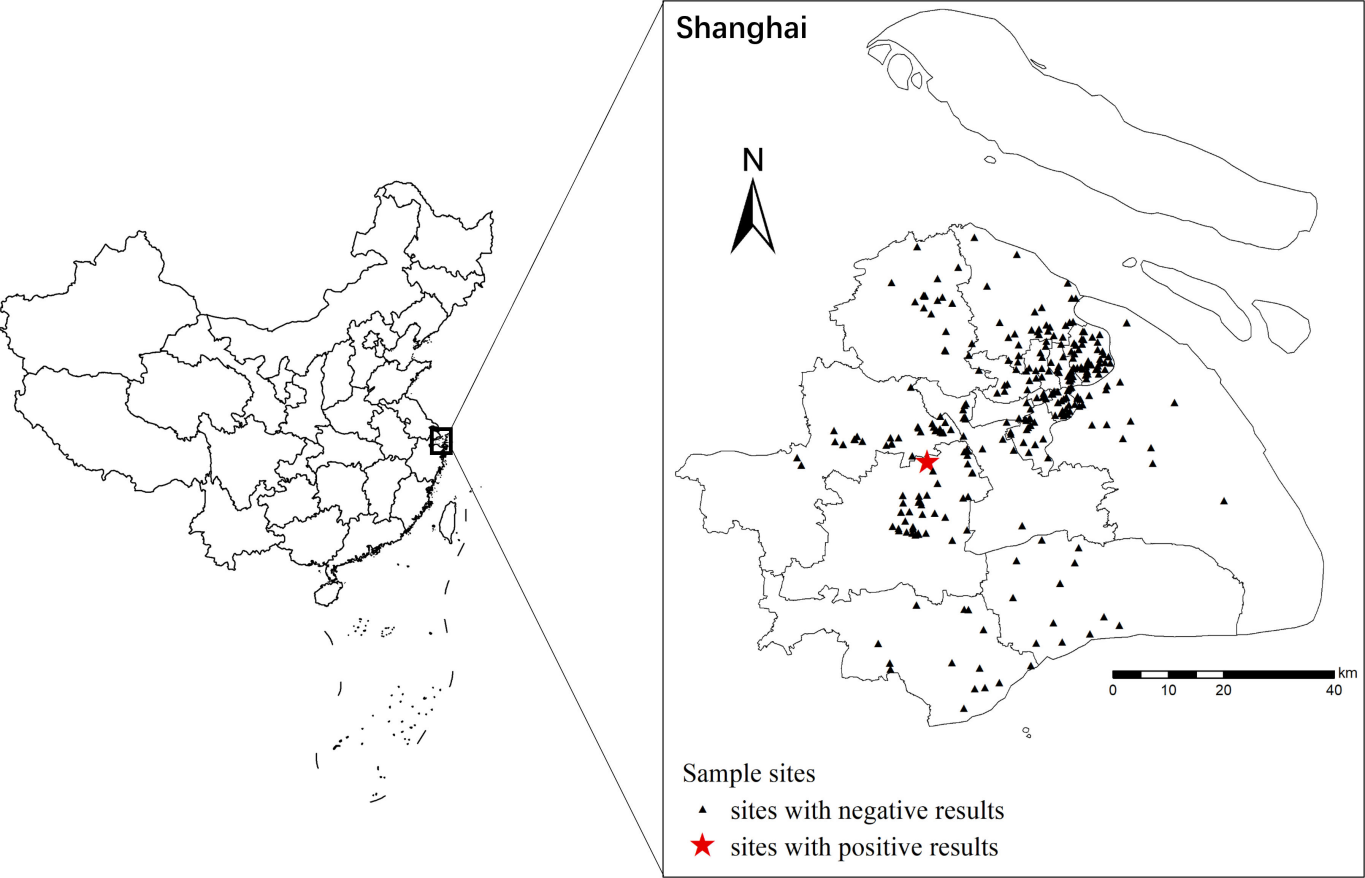
Geographical locations of the sample sites in this study. The sites with negative results were indicated with triangle, while sites with positive results were shown in red. The maps were created using ArcGIS 10.3 software.

Of the 306 *E. coli* isolates from hospital wastewater in 15 districts included in this study during May to August 2022, samples were mainly isolated from Songjiang (*n* = 41, 13.40%), Qingpu (*n* = 36, 11.76%), and Yangpu (*n* = 36, 11.76%) (Fig. 1). For the medical institutions, the samples were mainly isolated from private clinics (*n* = 196, 64.05%), followed by grade IIA hospitals (*n* = 54, 17.65%) and LTCFs (*n* = 23, 7.52%). There were significant differences observed on the samples among those facilities (*P* < 0.05). None of these isolates carried *mcr*-2 *to mcr*-10 gene; only the *E. coli* isolate ECSJ33 isolated from an LTCF located in Songjiang District carried the *mcr-1* gene.

### Susceptibility to antimicrobial and conjugative isolates

ECSJ33 exhibited colistin resistance at 4 µg/mL, which was considered as the resistance of *E. coli* according to EUCAST standards (Table 1). Furthermore, ECSJ33 was also found to be resistant to AMP, SXT, CHL, and TET. However, it was found to be susceptible to 15 other common antibiotics, including CIP, CTX, CZA, IMP, ETP, TIG, and others (as shown in Table 1). The result indicated that the *mcr-1*-harboring plasmid was capable of successful transfer from the donor strain to the recipient strain (*E. coli* C600). The conjugation of ECSJ33 to *E. coli* C600 via horizontal transfer was achieved with an average efficiency of 2.9 × 10^−2^. The transconjugant ECSJ33-T exhibited an MIC value of 4 µg/mL to colistin, which was increased when compared to the recipient *E. coli* C600 (0.25 µg/mL). That means ECSJ33-T acquired the colistin resistance gene from the donor strain.

**TABLE 1:** Antimicrobial susceptibility of the *mcr-1*-harboring *E. coli* SJ33 and its transconjugant ECSJ33-T^*a*^ identified in this study

Antibiotic	SJ33		ECSJ33-T		EC C600	
	MIC	Result	MIC	Result	MIC	Result
CIP	0.25	S	1	R	≤0.015	S
AMP	＞64	R	＞64	R	4	S
AMS	16/8	I	16/8	I	4/2	S
CT	4	I	4	I	0.25	S
CFZ	4	I	4	R	2	S
CTX	≤0.25	S	≤0.25	R	≤0.25	S
CAZ/C	≤0.25/4	−^*b*^	≤0.25/4	−	≤0.25/4	−
CTX/C	≤0.125/4	−	≤0.125/4	−	≤0.125/4	−
CFX	4	S	8	S	2	S
CPM	≤1	S	≤1	S	≤1	S
CXM	2	S	8	R	≤0.5	S
CZA	≤0.25/4	S	≤0.25/4	S	≤0.25/4	S
IMP	≤0.25	S	≤0.25	S	≤0.25	S
CAZ	≤0.25	S	≤0.25	S	0.5	S
AZI	≤2	−	≤2	−	≤2	−
ETP	≤0.25	S	≤0.25	S	≤0.25	S
SXT	＞8/152	R	＞8/152	R	＞8/152	R
NAL	16	S	＞64	R	＞64	R
CHL	＞64	R	＞64	R	4	S
GEN	≤1	S	≤1	R	≤1	S
TET	＞32	R	32	R	≤1	S
TIG	≤0.25	S	≤0.25	S	≤0.25	S
AMI	≤2	S	≤2	S	≤2	S
ATM	≤2	S	≤2	R	≤2	S
LEV	0.5	S	2	R	≤0.125	S
MEM	≤0.125	S	≤0.125	S	≤0.125	S
STR	≤4	−	≤4	−	8	−
NOR	1	S	4	R	≤0.125	S

^*a*^ R, resistant; I, intermediate; S, susceptible.

^*b*^ This could not provide the results for the MIC obtained in the study.

### Genetic characterization of *mcr-1*-harboring plasmids

**Fig 2:**
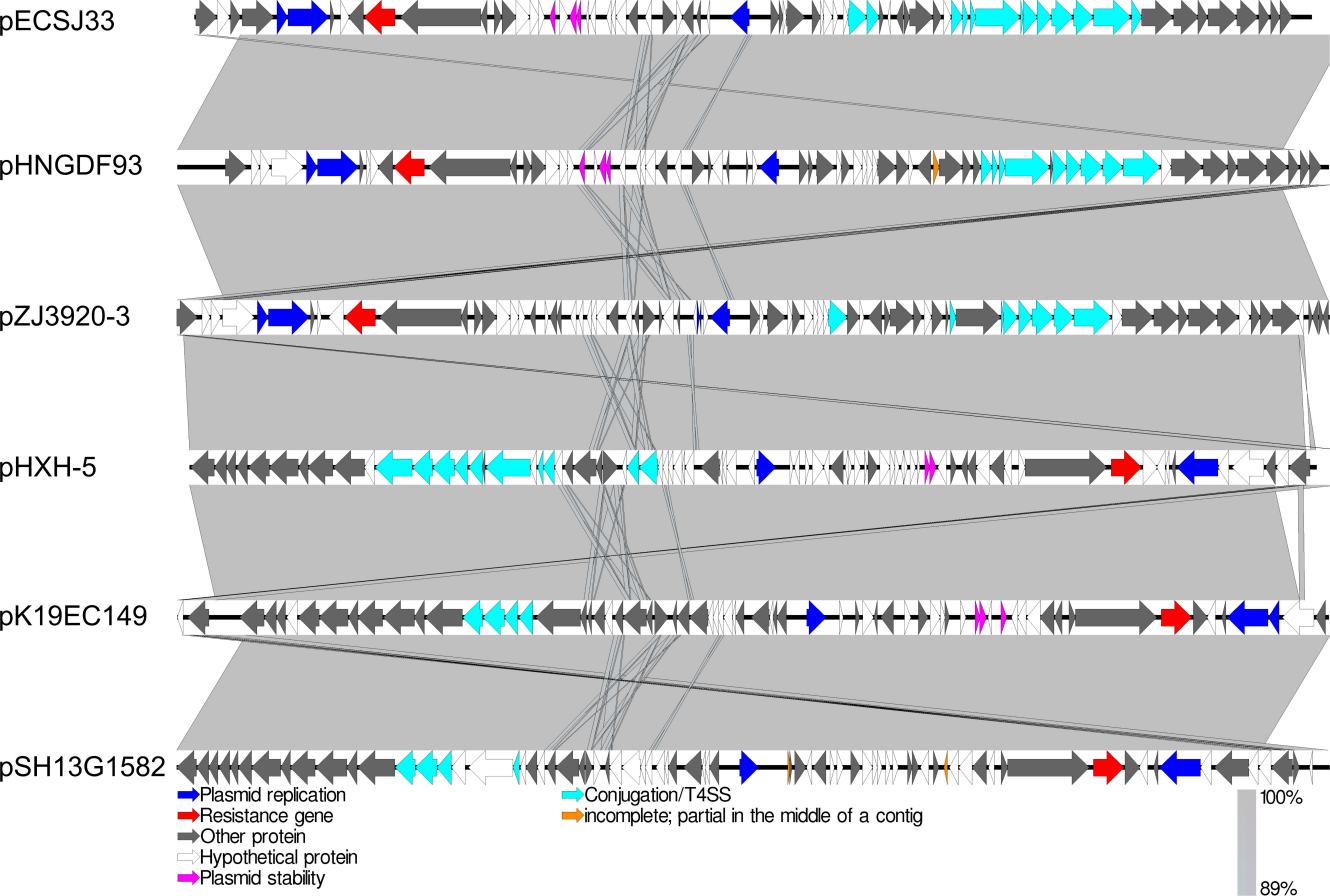
Linear comparison of complete plasmid sequences of plasmid *E. coli* pECSJ33 (this study), pHNGDF93 from *E. coli* GDT6F93 (GenBank accession no. MF978388), pHXH-5 from *E. coli* HXH-5 (GenBank accession no. MH202956), pK19EC149 from *E. coli* pK19EC149 (GenBank accession no. CP050290), pSH13G1582 from *Salmonella enterica* subsp. *enterica* serovar Typhimurium strain SH13G1582 (GenBank accession no. MH522412), and pZJ3920-3 from *E. coli* ZJ3920 (GenBank accession no. CP020548). The arrows represent the position and transcriptional direction of theopen reading frames (ORFs). Resistance gene (*mcr-1*) is indicated by the red arrow, plasmid replication proteins are indicated in blue, plasmid stability proteins are highlighted in pink arrows, and type IV secretion system genes are indicated by light blue arrows.

**Fig 3:**
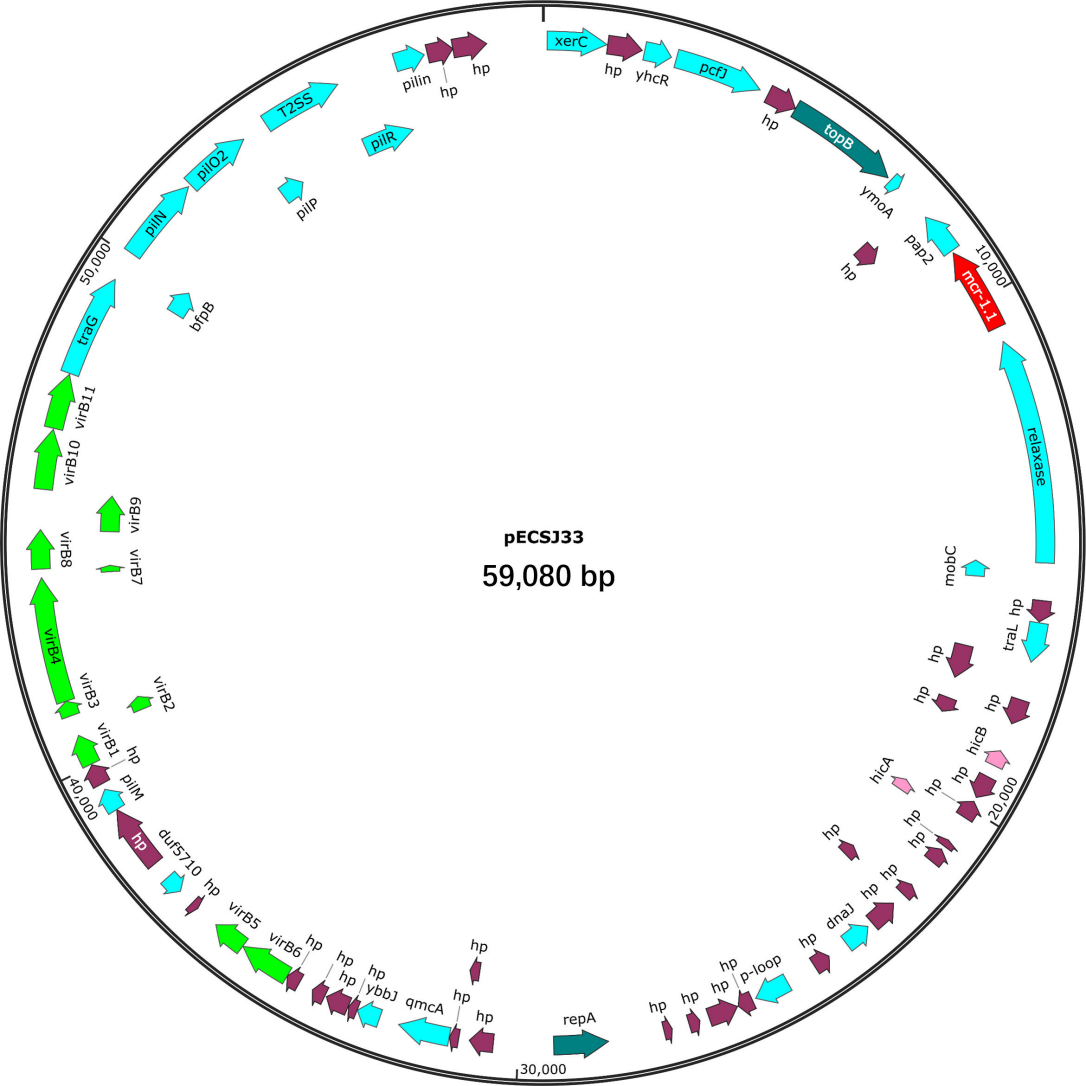
Map of *mcr-1*-harboring plasmid pECSJ33. The *mcr-1* gene is marked in red. The figure was created using SnapGene Viewer software.

Apart from *mcr-1*, ECSJ33 was also found to carry resistance genes for *bla*_TEM-135_ (β-lactamase), *dfrA*14 (trimethoprim-resistant dihydrofolate reductase), *qnrS*1 (quinolone-resistant protein), and other resistant genes relating to antibiotic efflux pump (*cpxA*, *emrR*, *emrB*, *acrE*, *tolC*, *msbA, evgA, mdtE*). The sequence type (ST) of ECSJ33 identified in this study was ST155. Whole-genome sequencing analysis revealed that the *mcr-1* gene was located on a 59,080 bp IncI2 plasmid in pECSJ33 isolate. BLASTn analysis showed that the backbone of the plasmid pECSJ33 (this study) was strikingly similar to (the query cover of 100% and the identities 99%) other previously sequenced *mcr-1*-harboring plasmids, such as pHNGDF93 from *E. coli* GDT6F93 (GenBank accession no. MF978388), pHXH-5 from *E. coli* HXH-5 (GenBank accession no. MH202956), pK19EC149 from *E. coli* pK19EC149 (GenBank accession no. CP050290), pSH13G1582 from *Salmonella enterica* subsp. *enterica* serovar Typhimurium strain SH13G1582 (GenBank accession no. MH522412), and pZJ3920-3 from *E. coli* ZJ3920 (GenBank accession no. CP020548). In all, the result showed that these *mcr-1*-harboring plasmids exhibited very high architectural conservation (>89% identity, Fig. 2). Furthermore, the BLAST comparison of these plasmids revealed that their *mcr-1* insertion sites differed: pECSJ33, pHNGDF93, and pZJ3920-3 shared the similar sites, while pHXH-5, pK19EC149, and pSH13G1582 had the same insertion sites (Fig. 2). An approximately 2.6 kb *mcr-1-pap2* element was identified in the above-mentioned plasmids. The putative conjugal transfer components of pECSJ33 were also detected by using oriTfinder. The *vir* gene family members encoding VirB1 to VirB11 were identified as belonging to the type IV secretion system (T4SS), as predicted on pECSJ33 (Fig. 3).

### Phylogenetic analysis

**Fig 4:**
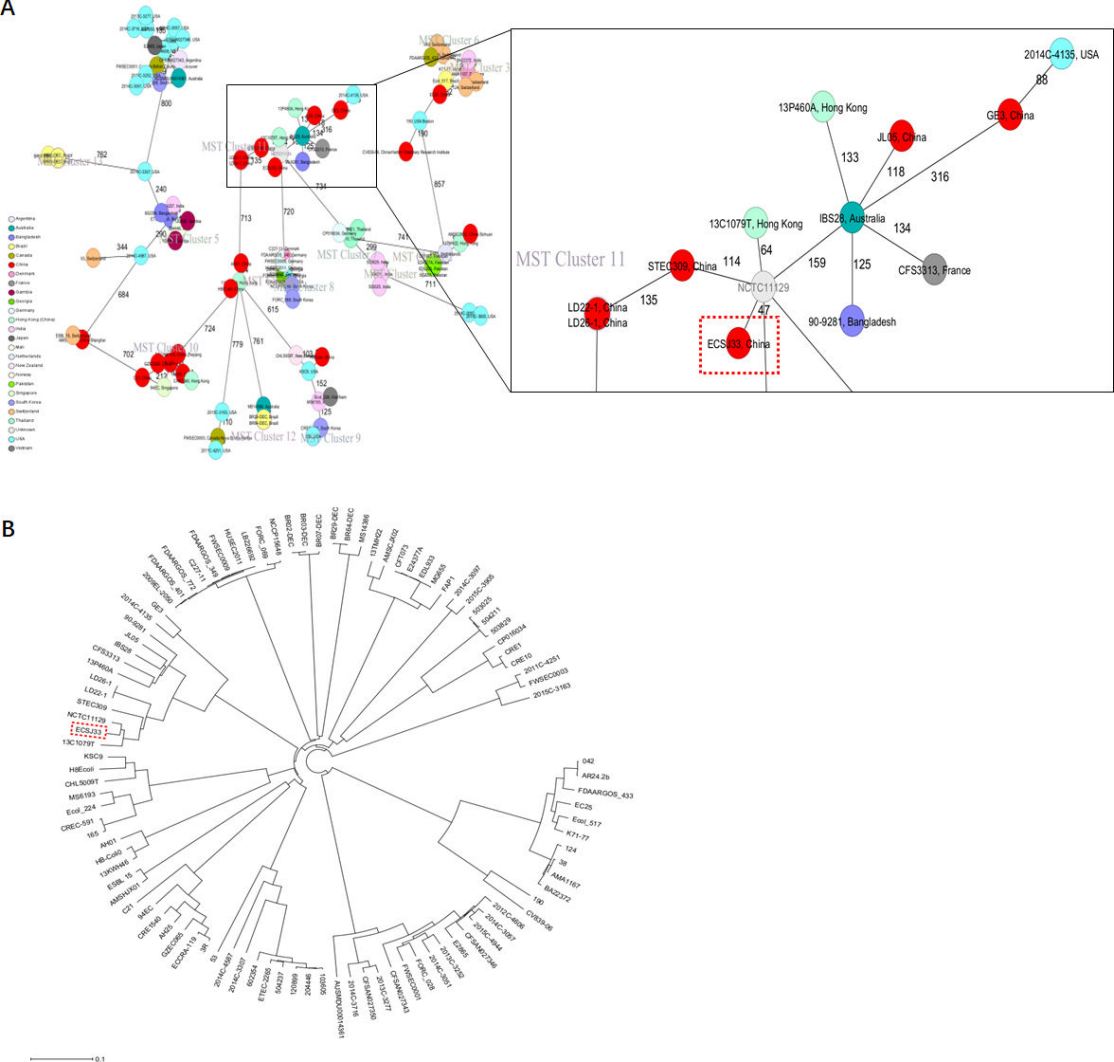
Phylogenetic analysis. (**A**) Minimum spanning tree constructed on the basis of cgMLST allelic genes of 101 MCRPEC strains. Each circle depicts an allelic profile based on sequence analysis of 3,179 cgMLST genes. The length of the connecting lines represents the number of target genes with different alleles. Each circle within the tree represents a cgMLST type, with diameters scaled to the number of isolates belonging to that type. Colors represent different isolation countries. Closely related genotypes (<10 alleles difference) are shaded in the same node, and clusters are numbered consecutively. (**B**) Maximum likelihood phylogenetic tree of 101 MCRPEC strains based on the core genome. The red dashed box indicates ECSJ33 strain.

The cgMLST analysis identified 3,179 core genes and was performed for the 101 *E. coli* isolates including ECSJ33. The minimal spanning tree (MST) showed strains with different sources, and 22 distinct clonal transmission events were observed across 101 genomes. ECSJ33 was found to be highly similar to NCTC11129 strain in cluster 11, with only 47 sites differing from each other (Fig. 4A). Strains clustered by cgMLST also revealed the deep branching and scattered population structure contained in distinct clades, which was broadly classified into distinct phylogenetic lineages (Fig. 4B).

**Fig 5:**
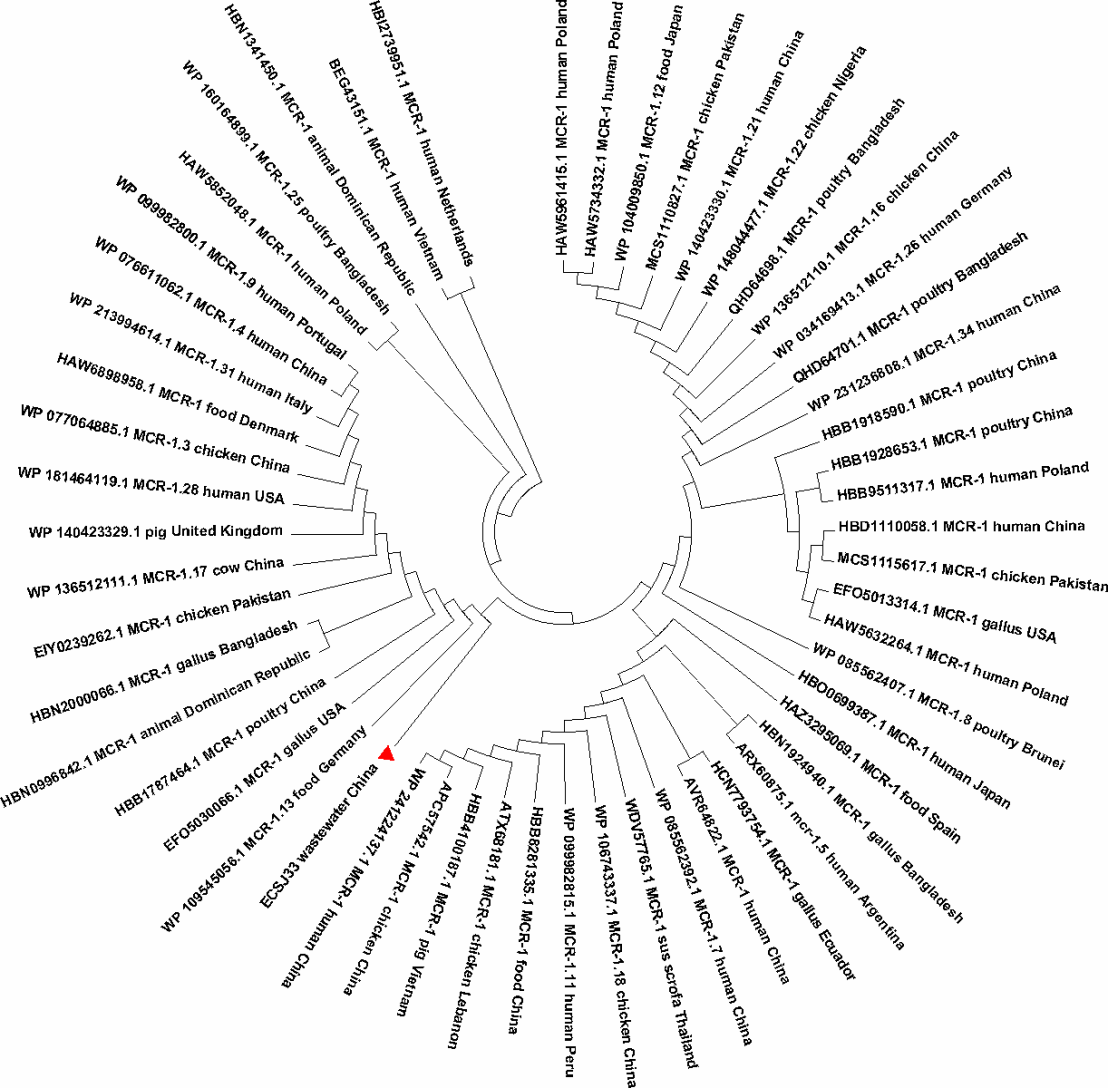
Phylogeny of 58 MCR-1 proteins of *E. coli* origin retrieved from the NCBI database. Using amino acid sequences from one *E. coli* MCR-1 protein in this study (ECSJ33), the BLAST search tool (https://blast.ncbi.nlm.nih.gov/Blast.cgi, accessed on 30 September 2023) was used to retrieve homologous sequences of MCR-1 and MCR-1-like proteins from the NCBI database. MCR-1 and MCR-1-like proteins of *E. coli* containing LptA and others were among the sequences categorized. Using aligned MCR-1 sequences from ClustalW, the maximum likelihood method of MEGA X was used to create a phylogenetic tree.

A total of 54 MCR-1 proteins that originated from *E. coli* (File S1) were categorized for further analysis. In the case of protein acquisition from the NCBI database, above 50% of query coverage was set as the screening point. The set of proteins (File S1), including ECSJ33 strain harboring MCR-1 in this study, was used for phylogenetic analysis (Fig. 5). The sequenced MCR-1 of ECSJ33 in this study clearly showed its genomic confirmation as *mcr-1* genes by highly aligning with *mcr-1* genes of *E. coli* as well as other bacterial origins. In addition, the phylogenetic tree showed that ECSJ33 strain in this study was mostly of animal food origin and that they were closely related.

## DISCUSSION

The emergence and spread of the *mcr-1* gene in *Enterobacteriaceae*, particularly *E. coli*, represents a significant challenge to public health worldwide. In this study, we report the detection of *mcr-1* in *E. coli* isolated from LTCF wastewater samples in Shanghai, China, highlighting the potential role of wastewater as a reservoir for antibiotic-resistant bacteria. The detection of *mcr-1* in LTCF wastewater samples is concerning, which is considered a last-resort antibiotic for the treatment of multidrug-resistant bacterial infections.

Our study suggests a lower prevalence (0.33%, 1/306) of *mcr-1* in LTCF wastewater than that reported from other LTCFs in other countries such as Italy (31) and the Netherlands (18). Furthermore, the prevalence was also lower than *E. coli* strains isolated from human populations in Shanghai (25, 32, 33), and it was not as high as that found in *E. coli* isolated from hospitalized companion animals (34). Given that those *E. coli* strain carriers were only detected during the prevalence survey, most carriers remain undetected in Shanghai.

One of the key findings of our study is the identification of a novel *mcr-1*-harboring plasmid, which we have designated pECSJ33. This plasmid was found in *E. coli* isolate from an LTCF in Songjiang District in Shanghai and was similar to the previously reported *mcr-1*-harboring pHNGDF93, which was isolated from fish in Guangdong Province. The phylogenetic tree showed that the MCR-1 protein of ECSJ33 was also found to be similar with MCR-1.13 isolated from food source in Germany (WP 109545655.1). Given this evidence, we speculated that ECSJ33 may have originated via the food chain and integrated into wastewater (8, 35, 36). This highlights the importance of surveillance efforts to monitor the emergence and dissemination of *mcr-1* among the elderly in LTCFs, as well as the co-existence of ESBL genes such as *bla*_TEM-135_ and *mcr-1* gene carrying *E. coli*, which have been reported globally, particularly in animal source *E. coli* isolates from food-producing animal in Poland located on IncX4 plasmid (37), from human in Thailand located on IncHI/IncN plasmid (38), and from human in China located on IncI2 plasmid (24). Our study identified, for the first time, the co-existence of *bla*_TEM-135_ and *mcr-1* from a wastewater source isolate in *E. coli* in Shanghai. This finding indicates a high risk of disseminating this extensively drug-resistant *E. coli*, which poses a threat to public health.

The finding of cgMLST and phylogenetic tree revealed that ECSJ33 isolate belonged to the ST155 that was similar to *E. coli* strain NCTC11129 (GenBank accession no. GCA_900636075.1); the latter was submitted by Wellcome Sanger Institute in 2014 with the accession no. PRJEB6403. However, the *mcr-1* gene was absent in NCTC11129, suggesting that the dissemination of *mcr-1* in the environment is likely due to the horizontal transfer of the IncI2 plasmid between different *E. coli* strains rather than clonal expansion of a single *mcr-1*-positive strain.

Previous studies have shown that wastewater samples like wastewater treatment plants can be a source of multidrug-resistant bacteria and antibiotic resistance genes in the environment (39, 40). Our study also demonstrates the potential for the spread of *mcr-1* from wastewater to human populations. In addition, untreated wastewater may contain high concentrations of antibiotic residues, providing a selective pressure for the emergence and dissemination of antibiotic-resistant bacteria (41). Therefore, it is important to develop strategies to mitigate the risk of transmission of antibiotic-resistant bacteria from wastewater to human populations.

There are two limitations to this study. Firstly, the *mcr-1* prevalence of *E. coli* isolates was only collected from wastewater samples in Shanghai, China. To obtain more accurate results, it would be beneficial to include isolates from additional sources including human, animal, and food, which could contribute to the dissemination of *mcr-1* transmission and spread using the “One Health” approach. Secondly, only one positive *E. coli* isolate carrying *mcr-1* gene was found in this study, which needs to be carried out among more isolates and from more regions.

### Conclusion

In conclusion, our study provides evidence for the prevalence and characteristics of *mcr-1*-positive *E. coli* isolated from wastewater of LTCFs in Shanghai, China. The *mcr-1* on an IncI2 plasmid *E. coli* strain found in our study was also detected to carry other multiple resistance genes, which highlights the potential for the spread of antibiotic resistance in the environment. This study underscores the need for continued surveillance and monitoring of resistance genes in wastewater and the environment, especially for LTCFs which were not paid important attention to in the past several years. Future research should focus on further characterizing the genetic and molecular mechanisms underlying the dissemination of *mcr-1* in wastewater from LTCFs and the environment, as well as on the development of novel strategies to mitigate the spread of antibiotic resistance.

## Data Availability

Sequences were deposited to the National Center for Biotechnology Information (NCBI) website under BioProject number PRJNA967846.

## SUPPLEMENTAL MATERIAL

Supplemental material spectrum.03707-23-s0001.docx Amino acid sequences of *E. coli* strains harboring the *mcr-1* gene in this study.

ASM does not own the copyrights to Supplemental Material that may be linked to, or accessed through, an article. The authors have granted ASM a non-exclusive, world-wide license to publish the Supplemental Material files. Please contact the corresponding author directly for reuse.
