# Supplementary material for: Genetic epidemiology and plasmid-mediated transmission of mcr-1 by Escherichia coli ST155 from wastewater of long-term care facilities
Source: Microbiol Spectr. 2024 Feb 14;12(3):e03707-23. doi: 10.1128/spectrum.03707-23 (PMC10913736; doi:10.1128/spectrum.03707-23)
Supplement: Supplemental material — Amino acid sequences of E. coli strains harboring the mcr-1 gene in this study. [file spectrum.03707-23-s0001.docx]

>ECSJ33 wastewater China

MMQHTSVWYRRSVSPFVLVASVAVFLTATANLTFFDKISQTYPIADNLGFVLTIAVVLFGAMLLITTLLSSYRYVLKPVLILLLIMGAVTSYFTDTYGTVYDTTMLQNALQTDQAETKDLLNAAFIMRIIGLGVLPSLLVAFVKVDYPTWGKGLMRRLGLIVASLALILLPVVAFSSHYASFFRVHKPLRSYVNPIMPIYSVGKLASIEYKKASAPKDTIYHAKDAVQATKPDMRKPRLVVFVVGETARADHVSFNGYERDTFPQLAKIDGVTNFSNVTSCGTSTAYSVPCMFSYLGADEYDVDTAKYQENVLDTLDRLGVSILWRDNNSDSKGVMDKLPKAQFADYKSATNNAICNTNPYNECRDVGMLVGLDDFVAANNGKDMLIMLHQMGNHGPAYFKRYDEKFAKFTPVCEGNELAKCEHQSLINAYDNALLATDDFIAQSIQWLQTHSNAYDVSMLYVSDHGESLGENGVYLHGMPNAFAPKEQRSVPAFFWTDKQTGITPMATDTVLTHDAITPTLLKLFDVTADKVKDRTAFIR

>HAW5852048.1 MCR-1 human Poland

MMQHTSVWYRRSVSPFVLVASVAVFLTATANLTFFDKISQTYPIADNLGFVLTIAVVLFGAMLLITTLLSSYRYVLKPVLILLLIMGAVTSYFTDTYGTVYDTTMLQNALQTDQAETKDLLNAAFIMRIIGLGVLPSLLVAFVKVDYPTWGKGLMRRLGLIVASLALILLPVVAFSSHYASFFRVHKPMRSYVNPIMPIYSVGKLASIEYKKASAPKDTIYHAKDAVQATKPDMRKPRLVVFVVGETARADHVSFNGYERDTFPQLAKIDGVTNFSNVTSCGTSTAYSVPCMFSYLGADEYDVDTAKYQENVLDTLDRLGVSILWRDNNSDSKGVMDKLPKAQFADYKSATNNAICNTNPYNECRDVGMLVGLDDFVAANNGKDMLIMLHQMGNHGPAYFKRYDEKFAKFTPVCEGNELAKCEHQSLINAYDNALLATDDFIAQSIQWLQTHSNAYDVSMLYVSDHGESLGENGVYLHGMPNAFAPKEQRSVPAFFWTDKQTGITPMATDTVLTHDAITPTLLKLFDVTADKVKDRTAFIR

>WP_136512111.1 MCR-1.17 cow China

MMQHTSVWYRRSVSPFVLVASVAVFLTATANLTFFDKISQTYPIADNLGFVLTIAVVLFGAMLLITTLLSSYRYVLKPVLILLLIMGAVTSYFTDTYGTVYDTTMLQNALQTDQAETKDLLNAAFIMRIIGLGVLPTLLVAFVKVDYPTWGKGLMRRLGLIVASLALILLPVVAFSSHYASFFRVHKPLRSYVNPIMPIYSVGKLASIEYKKASAPKDTIYHAKDAVQATKPDMRKPRLVVFVVGETARADHVSFNGYERDTFPQLAKIDGVTNFSNVTSCGTSTAYSVPCMFSYLGADEYDVDTAKYQENVLDTLDRLGVSILWRDNNSDSKGVMDKLPKAQFADYKSATNNAICNTNPYNECRDVGMLVGLDDFVAANNGKDMLIMLHQMGNHGPAYFKRYDEKFAKFTPVCEGNELAKCEHQSLINAYDNALLATDDFIAQSIQWLQTHSNAYDVSMLYVSDHGESLGENGVYLHGMPNAFAPKEQRSVPAFFWTDKQTGITPMATDTVLTHDAITPTLLKLFDVTADKVKDRTAFIR

>WP_140423329.1 pig United Kingdom

MMQHTSVWYRRSVSPFVLVASVAVFLTATANLTFFDKISQTYPIADNLGFVLTIAVVLFGALLLITTLLSSYRYVLKPVLILLLIMGAVTSYFTDTYGTVYDTTMLQNALQTDQAETKDLLNAAFIMRIIGLGVLPSLLVAFVKVDYPTWGKGLMRRLGLIVASLALILLPVVAFSSHYASFFRVHKPLRSYVNPIMPIYSVGKLASIEYKKASAPKDTIYHAKDAVQATKPDMRKPRLVVFVVGETARADHVSFNGYERDTFPQLAKIDGVTNFSNVTSCGTSTAYSVPCMFSYLGADEYDVDTAKYQENVLDTLDRLGVSILWRDNNSDSKGVMDKLPKAQFADYKSATNNAICNTNPYNECRDVGMLVGLDDFVAANNGKDMLIMLHQMGNHGPAYFKRYDEKFAKFTPVCEGNELAKCEHQSLINAYDNALLATDDFIAQSIQWLQTHSNAYDVSMLYVSDHGESLGENGVYLHGMPNAFAPKEQRSVPAFFWTDKQTGITPMATDTVLTHDAITPTLLKLFDVTADKVKDRTAFIR

>WP_085562392.1 MCR-1.7 human China

MMQHTSVWYRRSVSPFVLVASVAVFLTATANLTFFDKISQTYPIADNLGFVLTIAVVLFGAMLLITTLLSSYRYVLKPVLILLLIMGAVTSYFTDTYGTVYDTTMLQNALQTDQAETKDLLNAAFIMRIIGLGVLPSLLVAFVKVDYPTWGKGLMRRLGLIVASLALILLPVVAFSSHYASFFRVHKPLRSYVNPIMPIYSVGKLASIEYKKASTPKDTIYHAKDAVQATKPDMRKPRLVVFVVGETARADHVSFNGYERDTFPQLAKIDGVTNFSNVTSCGTSTAYSVPCMFSYLGADEYDVDTAKYQENVLDTLDRLGVSILWRDNNSDSKGVMDKLPKAQFADYKSATNNAICNTNPYNECRDVGMLVGLDDFVAANNGKDMLIMLHQMGNHGPAYFKRYDEKFAKFTPVCEGNELAKCEHQSLINAYDNALLATDDFIAQSIQWLQTHSNAYDVSMLYVSDHGESLGENGVYLHGMPNAFAPKEQRSVPAFFWTDKQTGITPMATDTVLTHDAITPTLLKLFDVTADKVKDRTAFIR

>HBO0699387.1 MCR-1 human Japan

MMQHTAVWYRRSVSPFVLVASVAVFLTATANLTFFDKISQTYPIADNLGFVLTIAVVLFGAMLLITTLLSSYRYVLKPVLILLLIMGAVTSYFTDTYGTVYDTTMLQNALQTDQAETKDLLNAAFIMRIIGLGVLPSLLVAFVKVDYPTWGKGLMRRLGLIVASLALILLPVVAFSSHYASFFRVHKPLRSYVNPIMPIYSVGKLASIEYKKASAPKDTIYHAKDAVQATKPDMRKPRLVVFVVGETARADHVSFNGYERDTFPQLAKIDGVTNFSNVTSCGTSTAYSVPCMFSYLGADEYDVDTAKYQENVLDTLDRLGVSILWRDNNSDSKGVMDKLPKAQFADYKSATNNAICNTNPYNECRDVGMLVGLDDFVAANNGKDMLIMLHQMGNHGPAYFKRYDEKFAKFTPVCEGNELAKCEHQSLINAYDNALLATDDFIAQSIQWLQTHSNAYDVSMLYVSDHGESLGENGVYLHGMPNAFAPKEQRSVPAFFWTDKQTGITPMATDTVLTHDAITPTLLKLFDVTADKVKDRTAFIR

>WP_181464119.1 MCR-1.28 human USA

MMQHTSVWYRRSVSPFVLVASVAVFLTATANLTFFDKISQTYPIADNLGFVLTIAVVLFGAMLLITTLLSSYRYVLKPVLILLLIMGAVTSYFTDTYGTVYDTTMLQNALQTDQAETKDLLNAAFIMRIIGLGVLPSLLVAFVKVDYPTWGKGLMRRLGLIVASLALILLPVVAFSSHYASFFRVHKPLRSYVNPIMPIYSVGKLASIEYKKANAPKDTIYHAKDAVQATKPDMRKPRLVVFVVGETARADHVSFNGYERDTFPQLAKIDGVTNFSNVTSCGTSTAYSVPCMFSYLGADEYDVDTAKYQENVLDTLDRLGVSILWRDNNSDSKGVMDKLPKAQFADYKSATNNAICNTNPYNECRDVGMLVGLDDFVAANNGKDMLIMLHQMGNHGPAYFKRYDEKFAKFTPVCEGNELAKCEHQSLINAYDNALLATDDFIAQSIQWLQTHSNAYDVSMLYVSDHGESLGENGVYLHGMPNAFAPKEQRSVPAFFWTDKQTGITPMATDTVLTHDAITPTLLKLFDVTADKVKDRTAFIR

>WP_077064885.1 MCR-1.3 chicken China

MMQHTSVWYRRSVSPFVLVASVAVFLTATANLTFFDKVSQTYPIADNLGFVLTIAVVLFGAMLLITTLLSSYRYVLKPVLILLLIMGAVTSYFTDTYGTVYDTTMLQNALQTDQAETKDLLNAAFIMRIIGLGVLPSLLVAFVKVDYPTWGKGLMRRLGLIVASLALILLPVVAFSSHYASFFRVHKPLRSYVNPIMPIYSVGKLASIEYKKASAPKDTIYHAKDAVQATKPDMRKPRLVVFVVGETARADHVSFNGYERDTFPQLAKIDGVTNFSNVTSCGTSTAYSVPCMFSYLGADEYDVDTAKYQENVLDTLDRLGVSILWRDNNSDSKGVMDKLPKAQFADYKSATNNAICNTNPYNECRDVGMLVGLDDFVAANNGKDMLIMLHQMGNHGPAYFKRYDEKFAKFTPVCEGNELAKCEHQSLINAYDNALLATDDFIAQSIQWLQTHSNAYDVSMLYVSDHGESLGENGVYLHGMPNAFAPKEQRSVPAFFWTDKQTGITPMATDTVLTHDAITPTLLKLFDVTADKVKDRTAFIR

>HAW6898958.1 MCR-1 food Denmark

MMQHTSVWYRRSVSPFVLVASVAVFLTATANLTFFDKISQTYPIADNLGFVLTIAVVLFGAMLLITTLLSSYRYVLKPVLILLLLMGAVTSYFTDTYGTVYDTTMLQNALQTDQAETKDLLNAAFIMRIIGLGVLPSLLVAFVKVDYPTWGKGLMRRLGLIVASLALILLPVVAFSSHYASFFRVHKPLRSYVNPIMPIYSVGKLASIEYKKASAPKDTIYHAKDAVQATKPDMRKPRLVVFVVGETARADHVSFNGYERDTFPQLAKIDGVTNFSNVTSCGTSTAYSVPCMFSYLGADEYDVDTAKYQENVLDTLDRLGVSILWRDNNSDSKGVMDKLPKAQFADYKSATNNAICNTNPYNECRDVGMLVGLDDFVAANNGKDMLIMLHQMGNHGPAYFKRYDEKFAKFTPVCEGNELAKCEHQSLINAYDNALLATDDFIAQSIQWLQTHSNAYDVSMLYVSDHGESLGENGVYLHGMPNAFAPKEQRSVPAFFWTDKQTGITPMATDTVLTHDAITPTLLKLFDVTADKVKDRTAFIR

>WP_213994614.1 MCR-1.31 human Italy

MMQHTSVWYRRSVSPFVLVASVAVFLTATANLTFFDKISQTYPIADNLGFVLTIAVVLFGAMLLITTLLSSYRYVLKPVLILLLIMGAVTSYFTDTYGTVYDTTMLQNALQTDQAETKDLLNAVFIMRIIGLGVLPSLLVAFVKVDYPTWGKGLMRRLGLIVASLALILLPVVAFSSHYASFFRVHKPLRSYVNPIMPIYSVGKLASIEYKKASAPKDTIYHAKDAVQATKPDMRKPRLVVFVVGETARADHVSFNGYERDTFPQLAKIDGVTNFSNVTSCGTSTAYSVPCMFSYLGADEYDVDTAKYQENVLDTLDRLGVSILWRDNNSDSKGVMDKLPKAQFADYKSATNNAICNTNPYNECRDVGMLVGLDDFVAANNGKDMLIMLHQMGNHGPAYFKRYDEKFAKFTPVCEGNELAKCEHQSLINAYDNALLATDDFIAQSIQWLQTHSNAYDVSMLYVSDHGESLGENGVYLHGMPNAFAPKEQRSVPAFFWTDKQTGITPMATDTVLTHDAITPTLLKLFDVTADKVKDRTAFIR

>WP_076611062.1 MCR-1.4 human China

MMQHTSVWYRRSVSPFVLVASVAVFLTATANLTFFDKISQTYPIADNLGFVLTIAVVLFGAMLLITTLLSSYRYVLKPVLILLLIMGAVTSYFTDTYGTVYDTTMLQNALQTDQAETKDLLNAAFIMRIIGLGVLPSLLVAFVKVDYPTWGKGLMRRLGLIVASLALILLPVVAFSSHYASFFRVHKPLRSYVNPIMPIYSVGKLASIEYKKASAPKDTIYHAKDAVQATKPDMRKPRLVVFVVGETARADHVSFNGYERDTFPQLAKIDGVTNFSNVTSCGTSTAYSVPCMFSYLGADEYDVDTAKYQENVLDTLDRLGVSILWRDNNSDSKGVMDKLPKAQFADYKSATNNAICNTNPYNECRDVGMLVGLDDFVAANNGKDMLIMLHQMGNHGPAYFKRYDEKFAKFTPVCEGNELAKCEHQSLINAYDNALLATDNFIAQSIQWLQTHSNAYDVSMLYVSDHGESLGENGVYLHGMPNAFAPKEQRSVPAFFWTDKQTGITPMATDTVLTHDAITPTLLKLFDVTADKVKDRTAFIR

>WP_099982800.1 MCR-1.9 human Portugal

MMQHTSVWYRRSVSPFVLVASVAVFLTATANLTFFDKISQTYPIADNLGFVLTIAVVLFGAMLLITTLLSSYRYVLKPVLILLLIMGAVTSYFTDTYGTVYDTTMLQNALQTDQAETKDLLNAAFIMRIIGLGVLPSLLVAFVKVDYPTWGKGLMRRLGLIVASLALILLPVVAFSSHYASFFRVHKPLRSYVNPIMPIYSVGKLASIEYKKASAPKDTIYHAKDAVQATKPDMRKPRLVVFVVGETARADHVSFNGYERDTFPQLAKIDGVTNFSNVTSCGTSTAYSVPCMFSYLGADEYDVDTAKYQENVLDTLDRLGVSILWRDNNSDSKGVMDKLPKAQFADYKSATNNAICNTNPYNECRDVGMLVGLDDFVAANNGKDMLIMLHQMGNHGPAYFKRYDEKFAKFTPACEGNELAKCEHQSLINAYDNALLATDDFIAQSIQWLQTHSNAYDVSMLYVSDHGESLGENGVYLHGMPNAFAPKEQRSVPAFFWTDKQTGITPMATDTVLTHDAITPTLLKLFDVTADKVKDRTAFIR

>BEG43151.1 MCR-1 human Vietnam

MMQHTSVWYRRSVSPFVLVASVAVFLTATANLTFFDKISQTYPIADNLGFVLTIAVVLFGAMLLITTLLSSYRYVLKPVLILLLIMGAVTSYFTDTYGTVYDTTMLQNALQTDQAETKDLLNAAFIMRIIGLGVLPSLLVAFVKVDYPTWGKGLMRRLGLIVASLALILLPVVAFSSHYASFFRVHKPLRSYVNPIMPIYSVGKLASIEYKKASAPKDTIYHAKDAVQATKPDMRKPRLVVFVVGETARADHVSFNGYERDTFPQLAKIDGVTNFSNVTSCGTSTAYSVPCMFSYLGADEYDVDTAKYQENVLDTLDRLGVSILWRDNNSNSKGVMDKLPKAQFADYKSATNNAICNTNPYNECRDVGMLVGLDDFVAANNGKDMLIMLHQMGNHGPAYFKRYDEKFAKFTPVCEGNELAKCEHQSLINAYDNALLATDDFIAQSIQWLQTHSNAYDVSMLYVSDHGESLGENGVYLHGMPNAFAPKEQRSVPAFFWTDKQTGITPMATDTVLTHDAITPTLLKLFDVTADKVKDRTAFIR

>EIY0239262.1 MCR-1 chicken Pakistan

MMQHTSVWYRRSVSPFVLVASVAVFLTATANLTFFDKISQTYPIADNLGFVLTIAVVLFGAMLLITTLLSSYRYVLKPVLILLLIMGAVTSYFTDTYGTVYDTTMLQNALQTDQAETKDLLNAAFIMRIIGLGVLPSLLVAFVKVDYPTWGKGLMRRLGLIVASLALILLPVVAFSSHYASFFRVHKPLRSYVNPIMPIYSVGKLASIEYKKASAPKDTIYHAKDAVQATKPDMRKPRLVVFVVGETARADHVSFNGYERDTFPQLAKIDGVTNFSNVTSCGTSTAYSVPCMFSYLGADEYDVDTAKYQENVLDTLDRLGVSILWRDNNSDSKGVMDKLPKAKFADYKSATNNAICNTNPYNECRDVGMLVGLDDFVAANNGKDMLIMLHQMGNHGPAYFKRYDEKFAKFTPVCEGNELAKCEHQSLINAYDNALLATDDFIAQSIQWLQTHSNAYDVSMLYVSDHGESLGENGVYLHGMPNAFAPKEQRSVPAFFWTDKQTGITPMATDTVLTHDAITPTLLKLFDVTADKVKDRTAFIR

>WP_160164899.1 MCR-1.25 poultry Bangladesh

MMQHTSVWYRRSVTPFVLVASVAVFLTATANLTFFDKISQTYPIADNLGFVLTIAVVLFGAMLLITTLLSSYRYVLKPVLILLLIMGAVTSYFTDTYGTVYDTTMLQNALQTDQAETKDLLNAAFIMRIIGLGVLPSLLVAFVKVDYPTWGKGLMRRLGLIVASLALILLPVVAFSSHYASFFRVHKPVRSYVNPIMPIYSVGKLASIEYKKASAPKDTIYHAKDAVQATKPDMRKPRLVVFVVGETARADHVSFNGYERDTFPQLAKIDGVTNFSNVTSCGTSTAYSVPCMFSYLGADEYDVDTAKYQENVLDTLDRLGVSILWRDNNSDSKGVMDKLPKAQFADYKSATNNAICNTNPYNECRDVGMLVGLDDFVAANNGKDMLIMLHQMGNHGPAYFKRYDEKFAKFTPVCEGNELAKCEHQSLINAYDNALLATDDFIAQSIQWLQTHSNAYDVSMLYVSDHGESLGENGVYLHGMPNAFAPKEQRSVPAFFWTDKQTGITPMATDTVLTHDAITPTLLKLFDVTADKVKDRTAFIR

>WP_085562407.1 MCR-1.8 poultry Brunei

MMRHTSVWYRRSVSPFVLVASVAVFLTATANLTFFDKISQTYPIADNLGFVLTIAVVLFGAMLLITTLLSSYRYVLKPVLILLLIMGAVTSYFTDTYGTVYDTTMLQNALQTDQAETKDLLNAAFIMRIIGLGVLPSLLVAFVKVDYPTWGKGLMRRLGLIVASLALILLPVVAFSSHYASFFRVHKPLRSYVNPIMPIYSVGKLASIEYKKASAPKDTIYHAKDAVQATKPDMRKPRLVVFVVGETARADHVSFNGYERDTFPQLAKIDGVTNFSNVTSCGTSTAYSVPCMFSYLGADEYDVDTAKYQENVLDTLDRLGVSILWRDNNSDSKGVMDKLPKAQFADYKSATNNAICNTNPYNECRDVGMLVGLDDFVAANNGKDMLIMLHQMGNHGPAYFKRYDEKFAKFTPVCEGNELAKCEHQSLINAYDNALLATDDFIAQSIQWLQTHSNAYDVSMLYVSDHGESLGENGVYLHGMPNAFAPKEQRSVPAFFWTDKQTGITPMATDTVLTHDAITPTLLKLFDVTADKVKDRTAFIR

>HBN0996842.1 MCR-1 animal Dominican Republic

MMQHTSVWYRRSVSPFVLVASVAVFLTATANLTFFDKISQTYPIADNLGLVLTIAVVLFGAMLLITTLLSSYRYVLKPVLILLLIMGAVTSYFTDTYGTVYDTTMLQNALQTDQAETKDLLNAAFIMRIIGLGVLPSLLVAFVKVDYPTWGKGLMRRLGLIVASLALILLPVVAFSSHYASFFRVHKPLRSYVNPIMPIYSVGKLASIEYKKASAPKDTIYHAKDAVQATKPDMRKPRLVVFVVGETARADHVSFNGYERDTFPQLAKIDGVTNFSNVTSCGTSTAYSVPCMFSYLGADEYDVDTAKYQENVLDTLDRLGVSILWRDNNSDSKGVMDKLPKAQFADYKSATNNAICNTNPYNECRDVGMLVGLDDFVAANNGKDMLIMLHQMGNHGPAYFKRYDEKFAKFTPVCEGNELAKCEHQSLINAYDNALLATDDFIAQSIQWLQTHSNAYDVSMLYVSDHGESLGENGVYLHGMPNAFAPKEQRSVPAFFWTDKQTGITPMATDTVLTHDAITPTLLKLFDVTADKVKDRTAFIR

>HBB1928653.1 MCR-1 poultry China

MMQHTSVWYRRSVSPFVLVASVAVFLTATANLTFFDKISQTYPIADNLGFVLTIAVVLFGAMLLITTLLSSYRYVLKPVLILLLIMGAVTSYFTDTYGTVYDTTMLQNALQTDQAETKDLLNAAFIMRIIGLGVLPSLLVAFVKVDYPTWGKGLMRRLGLIVASLALILLPVVAFSSHYASFFRVHKPLRSYVNPIMPIYSVGKLASIEYKKASAPKDTIYHAKDAVQATKPDMRKPRLVVFVVGETARADHVSFNGYERDTFPQLAKIDGVTNFSNVTSCGTSTAYSVPCMFSYLGADEYDVDTAKYQENVLDTLDRLGVSILWRDNNSDSKGVMDKLPKAQFADYKSATNNAICNTNPYNECRDVGMLVGLDDFVAANNGKDMLIMLHQMGNHGPAYFKRYDEKFAKFTPVCEGNELAKCEHQSLINAYDNALLATDDFIAQSIQWLQTHSNAYDVSMLYVSDHGESLGENGVYLHGMPNAFAPKEQRSVPALFWTDKQTGITPMATDTVLTHDAITPTLLKLFDVTADKVKDRTAFIR

>HBN2000066.1 MCR-1 gallus Bangladesh

MMQHTSVWYRRSVSPFVLVASVAVFLTATANLTFFDKISQTYPIADNLGFVLTIAVVRFGAMLLITTLLSSYRYVLKPVLILLLIMGAVTSYFTDTYGTVYDTTMLQNALQTDQAETKDLLNAAFIMRIIGLGVLPSLLVAFVKVDYPTWGKGLMRRLGLIVASLALILLPVVAFSSHYASFFRVHKPLRSYVNPIMPIYSVGKLASIEYKKASAPKDTIYHAKDAVQATKPDMRKPRLVVFVVGETARADHVSFNGYERDTFPQLAKIDGVTNFSNVTSCGTSTAYSVPCMFSYLGADEYDVDTAKYQENVLDTLDRLGVSILWRDNNSDSKGVMDKLPKAQFADYKSATNNAICNTNPYNECRDVGMLVGLDDFVAANNGKDMLIMLHQMGNHGPAYFKRYDEKFAKFTPVCEGNELAKCEHQSLINAYDNALLATDDFIAQSIQWLQTHSNAYDVSMLYVSDHGESLGENGVYLHGMPNAFAPKEQRSVPAFFWTDKQTGITPMATDTVLTHDAITPTLLKLFDVTADKVKDRTAFIR

>WP_231236808.1 MCR-1.34 human China

MIQHTSVWYRRSVSPFVLVASVAVFLTATANLTFFDKISQTYPIADNLGFVLTIAVVLFGAMLLITTLLSSYRYVLKPVLILLLIMGAVTSYFTDTYGTVYDTTMLQNALQTDQAETKDLLNAAFIMRIIGLGVLPSLLVAFVKVDYPTWGKGLMRRLGLIVASLALILLPVVAFSSHYASFFRVHKPLRSYVNPIMPIYSVGKLASIEYKKASAPKDTIYHAKDAVQATKPDMRKPRLVVFVVGETARADHVSFNGYERDTFPQLAKIDGVTNFSNVTSCGTSTAYSVPCMFSYLGADEYDVDTAKYQENVLDTLDRLGVSILWRDNNSDSKGVMDKLPKAQFADYKSATNNAICNTNPYNECRDVGMLVGLDDFVAANNGKDMLIMLHQMGNHGPAYFKRYDEKFAKFTPVCEGNELAKCEHQSLINAYDNALLATDDFIAQSIQWLQTHSNAYDVSMLYVSDHGESLGENGVYLHGMPNAFAPKEQRSVPAFFWTDKQTGITPMATDTVLTHDAITPTLLKLFDVTADKVKDRTAFIR

>HBD1110058.1 MCR-1 human China

MMQHTSVWYRRSVSPFVLVASVAVFLTATANLTFFDKISQTYPIADNLGFVLTIAVVLFGAMLLITTLLSSYRYVLKPVLILLLIMGAVTSYFTDTYGTVYDTTMLQNALQTAQAETKDLLNAAFIMRIIGLGVLPSLLVAFVKVDYPTWGKGLMRRLGLIVASLALILLPVVAFSSHYASFFRVHKPLRSYVNPIMPIYSVGKLASIEYKKASAPKDTIYHAKDAVQATKPDMRKPRLVVFVVGETARADHVSFNGYERDTFPQLAKIDGVTNFSNVTSCGTSTAYSVPCMFSYLGADEYDVDTAKYQENVLDTLDRLGVSILWRDNNSDSKGVMDKLPKAQFADYKSATNNAICNTNPYNECRDVGMLVGLDDFVAANNGKDMLIMLHQMGNHGPAYFKRYDEKFAKFTPVCEGNELAKCEHQSLINAYDNALLATDDFIAQSIQWLQTHSNAYDVSMLYVSDHGESLGENGVYLHGMPNAFAPKEQRSVPAFFWTDKQTGITPMATDTVLTHDAITPTLLKLFDVTADKVKDRTAFIR

>HBB1787464.1 MCR-1 poultry China

MMQHTSVWYRRSVSPFVLVASVAVFLTATANLTFFDKISQTYPIADNLGFVLTIAVVLFGAMLLITTLLSSYRYVLKPVLILLLIMGAVTSYFTDTYGTVYDTTMLQNALQTDQAETKDLLNAAFIMRIIGLGVLPSLLVAFVKVDYPTWGKGLMRRLGLIVASLALILLPVVAFSSHYASFFRVHKPLRSYVNPIMPIYSVGKLASIEYKKASAPKDTIYHAKDAVQATKPDMRKPRLVVFVVGETARADHVSFNGYERDTFPQLAKIDGVTNFSNVTSCGTSTAYSVPCMFSYLGADEYDVDTAKYQENVLDTLDRLGVSILWRDNHSDSKGVMDKLPKAQFADYKSATNNAICNTNPYNECRDVGMLVGLDDFVAANNGKDMLIMLHQMGNHGPAYFKRYDEKFAKFTPVCEGNELAKCEHQSLINAYDNALLATDDFIAQSIQWLQTHSNAYDVSMLYVSDHGESLGENGVYLHGMPNAFAPKEQRSVPAFFWTDKQTGITPMATDTVLTHDAITPTLLKLFDVTADKVKDRTAFIR

>EFO5030066.1 MCR-1 gallus USA

MMQHTSVWYRRSVSPFVLVASVAVFLTATANLTFFDKISQTYPIADNLGFVLTIAVVLFGAMLLITTLLSSYRYVLKPVLILLLIMGAVTSYFTDTYGTVYDTTMLQNALQTDQAETKDLLNAAFIMRIIGLGVLPSLLVAFVKVDYPTWGKGLMRRLGLIVASLALILLPVVAFSSHYASFFRVHKPLRSYVNPIMPIYSVGKLASIEYKKASAPKDTIYHAKDAVQATKPDMRKPRLVVFVVGETARADHVSFNGYERDTFPQRAKIDGVTNFSNVTSCGTSTAYSVPCMFSYLGADEYDVDTAKYQENVLDTLDRLGVSILWRDNNSDSKGVMDKLPKAQFADYKSATNNAICNTNPYNECRDVGMLVGLDDFVAANNGKDMLIMLHQMGNHGPAYFKRYDEKFAKFTPVCEGNELAKCEHQSLINAYDNALLATDDFIAQSIQWLQTHSNAYDVSMLYVSDHGESLGENGVYLHGMPNAFAPKEQRSVPAFFWTDKQTGITPMATDTVLTHDAITPTLLKLFDVTADKVKDRTAFIR

>WP_109545056.1 MCR-1.13 food Germany

MMQHTSVWYRRSVSPFVLVASVAVFLTATANLTFFDKISQTYPIADNLGFVLTIAVVLFGAMLLITTLLSSYRYVLKPVLILLLIMGAVTSYFTDTYGTVYDTTMLQNALQTDQAETKDLLNAAFIMRIIGLGVLPSLLVAFVKVDYPTWGKGLIRRLGLIVASLALILLPVVAFSSHYASFFRVHKPLRSYVNPIMPIYSVGKLASIEYKKASAPKDTIYHAKDAVQATKPDMRKPRLVVFVVGETARADHVSFNGYERDTFPQLAKIDGVTNFSNVTSCGTSTAYSVPCMFSYLGADEYDVDTAKYQENVLDTLDRLGVSILWRDNNSDSKGVMDKLPKAQFADYKSATNNAICNTNPYNECRDVGMLVGLDDFVAANNGKDMLIMLHQMGNHGPAYFKRYDEKFAKFTPVCEGNELAKCEHQSLINAYDNALLATDDFIAQSIQWLQTHSNAYDVSMLYVSDHGESLGENGVYLHGMPNAFAPKEQRSVPAFFWTDKQTGITPMATDTVLTHDAITPTLLKLFDVTADKVKDRTAFIR

>QHD64701.1 MCR-1 poultry Bangladesh

MMQHTSVWYRRSVSPFVLVASVAVFLTATANLTFFDKISQTYPIADNLGFVLTIAVVLFGAMLLITTLLSSYRYVLKPVLILLLIMGAVTSYFTDTYGTVYDTTMLQNALQTDQAETKDLLNAAFIMRIIGLGVLPSLLVAFVKVDYPTWGKGLMRRLGLIVASLALILLPVVAFSSHYASFFRVHKPLRSYVNPIMPIYSVGKLASIEYKKASAPKDTIYHAKDAVQATKPDMRKPRLVVFVVGETARADHVSFNGYERDTFPQLAKIDGVTNFSNVTSCGTSTAYSVPCMFSYLGADEYDVDTAKYQENVLDTLDRLGVSILWRDNNSDSKGVMDKLPKAQFADYKSATNNAICNTNPYNECRDVGMLVGLDDFVAANNGKDMLIMLHQMGNHGPAYFKRYDEKFAKFTPVCEGNELAKCEHQSLINAYDNALLATDDFIAQSIQWLQTHSNAYDVSMLYVSDHGESLGENGVYLHGMPNAFAPKEQRSVPAFFWTDKQTGITPMATDTVLTHDAITPTLLKLFDVTADKVKDRTAXIR

>HBN1341450.1 MCR-1 animal Dominican Republic

MMQHTSVWYRRSVSPFVLVASVAVFLTATANLTFFDKISQTYPIADNLGFVLTIAVVLFGAMLLITTLLSSYRYVLKPVLILLLIMGAVTSYFTDTYGTVYDTTMLQNALQTDQAETKDLLNAAFIMRIIGLGVLPSLLVAFVKVDYPTWGKGLMRRLGLIVASLALILLPVVAFSSHYASFFRVHKPLRSYVNPIMPIYSVGKLASIEYKKASAPKDTIYHAKDAVQATKPDMRKPRLVVFVVGETARADHVSFNGYERDTFPQLAKIDGVTNFSNVTSCGTSTAYSVPCMFSYLGADEYDVDTAKYQENVLDTLDRLGVSILWRDNNSDSKGVMDKLPKAQFADYKSATNNAICNTNPYNECHDVGMLVGLDDFVAANNGKDMLIMLHQMGNHGPAYFKRYDEKFAKFTPVCEGNELAKCEHQSLINAYDNALLATDDFIAQSIQWLQTHSNAYDVSMLYVSDHGESLGENGVYLHGMPNAFAPKEQRSVPAFFWTDKQTGITPMATDTVLTHDAITPTLLKLFDVTADKVKDRTAFIR

>WP_034169413.1 MCR-1.26 human Germany

MQHTSVWYRRSVSPFVLVASVAVFLTATANLTFFDKISQTYPIADNLGFVLTIAVVLFGAMLLITTLLSSYRYVLKPVLILLLIMGAVTSYFTDTYGTVYDTTMLQNALQTDQAETKDLLNAAFIMRIIGLGVLPSLLVAFVKVDYPTWGKGLMRRLGLIVASLALILLPVVAFSSHYASFFRVHKPLRSYVNPIMPIYSVGKLASIEYKKASAPKDTIYHAKDAVQATKPDMRKPRLVVFVVGETARADHVSFNGYERDTFPQLAKIDGVTNFSNVTSCGTSTAYSVPCMFSYLGADEYDVDTAKYQENVLDTLDRLGVSILWRDNNSDSKGVMDKLPKAQFADYKSATNNAICNTNPYNECRDVGMLVGLDDFVAANNGKDMLIMLHQMGNHGPAYFKRYDEKFAKFTPVCEGNELAKCEHQSLINAYDNALLATDDFIAQSIQWLQTHSNAYDVSMLYVSDHGESLGENGVYLHGMPNAFAPKEQRSVPAFFWTDKQTGITPMATDTVLTHDAITPTLLKLFDVTADKVKDRTAFIR

>WP_136512110.1 MCR-1.16 chicken China

MMQHTSVWYRRSVSPFVLVASVAVFLTATANLTFFDKISQTYPIADNLGFVLTIAVVLFGAMLLITTLLSSYRYVLKPVLILLLIMGAVTSYFTDTYGTVYDTTMLQNALQTDQAETKDLLNAAFIMRIIGLGVLPSLLVAFVKVDYPTWGKGLMRRLGLIVASLALILLPVVAFSSHYASFFRVHKPLRSYVNPIMPIYSVGKLASIEYKKASAPKDTIYHAKDAVQATKPDMRKPRLVVFVVGETARADHVSFNGYERDTFPQLAKIDGVTNFSNVTSCGTSTAYSVPCMFSYLGADEYDVDTAKYQENVLDTLDSLGVSILWRDNNSDSKGVMDKLPKAQFADYKSATNNAICNTNPYNECRDVGMLVGLDDFVAANNGKDMLIMLHQMGNHGPAYFKRYDEKFAKFTPVCEGNELAKCEHQSLINAYDNALLATDDFIAQSIQWLQTHSNAYDVSMLYVSDHGESLGENGVYLHGMPNAFAPKEQRSVPAFFWTDKQTGITPMATDTVLTHDAITPTLLKLFDVTADKVKDRTAFIR

>QHD64698.1 MCR-1 poultry Bangladesh

MMQHTSVWYRRSVSPFVLVASVAVFLTATANLTFFDKISQTYPIADNLGFVLTIAVVLFGAMLLITTLLSSYRYVLKPVLILLLIMGAVTSYFTDTYGTVYDTTMLQNALQTDQAETKDLLNAAFIMRIIGLGVLPSLLVAFVXVDYPTWGKGLMRRLGLIVASLALILLPVVAFSSHYASFFRVHKPLRSYVNPIMPIYSVGKLASIEYKKASAPKDTIYHAKDAVQATKPDMRKPRLVVFVVGETARADHVSFNGYERDTFPQLAKIDGVTNFSNVTSCGTSTAYSVPCMFSYLGADEYDVDTAKYQENVLDTLDRLGVSILWRDNNSDSKGVMDKLPKAQFADYKSATNNAICNTNPYNECRDVGMLVGLDDFVAANNGKDMLIMLHQMGNHGPAYFKRYDEKFAKFTPVCEGNELAKCEHQSLINAYDNALLATDDFIAQSIQWLQTHSNAYDVSMLYVSDHGESLGENGVYLHGMPNAFAPKEQRSVPAFFWTDKQTGITPMATDTVLTHDAITPTLLKLFDVTADKVKDRTAFIR

>WP_148044477.1 MCR-1.22 chicken Nigeria

MMQHTSVWYRRSVSPFVLVASVAVFLTATANLTFFDKISQTYPIADNLGFVLTIAVVLFGAMLLITTLLSSYRYVLKPVLILLLIMGAVTSYFTDTYGTVYDTTMLQNALQTDQAETKDLLNAAFIMRIIGLGVLPSLLVAFVKVDYPTWGKGLMRRLGLIVASLALILLPVVAFSSHYASFFRVHKPLRSYVNPIMPIYSVGKLASIEYKKASAPKDTIYHAKDAVQATKPDMRKPRLVVFVVGETARADHVSFNGYERDTFPQLAKIDGVTNFSNVTSCGTSTAYSVPCMFSYLGADEYDVDTAKYQENVLDTLDRLGVSILWRDNNSDSKGVMDKLPKAQFADYKSATNNAICNTNPYNECRDVGMLVGLDDFVAANNGKDMLIMLHQMGNHGPAYFKRYDEKFAKFTPVCEGNELAKCEHQFLINAYDNALLATDDFIAQSIQWLQTHSNAYDVSMLYVSDHGESLGENGVYLHGMPNAFAPKEQRSVPAFFWTDKQTGITPMATDTVLTHDAITPTLLKLFDVTADKVKDRTAFIR

>WP_104009850.1 MCR-1.12 food Japan

MMHHTSVWYRRSVSPFVLVASVAVFLTATANLTFFDKISQTYPIADNLGFVLTIAVVLFGAMLLITTLLSSYRYVLKPVLILLLIMGAVTSYFTDTYGTVYDTTMLQNALQTDQAETKDLLNAAFIMRIIGLGVLPSLLVAFVKVDYPTWGKGLMRRLGLIVASLALILLPVVAFSSHYASFFRVHKPLRSYVNPIMPIYSVGKLASIEYKKASAPKDTIYHAKDAVQATKPDMRKPRLVVFVVGETARADHVSFNGYERDTFPQLAKIDGVTNFSNVTSCGTSTAYSVPCMFSYLGADEYDVDTAKYQENVLDTLDRLGVSILWRDNNSDSKGVMDKLPKAQFADYKSATNNAICNTNPYNECRDVGMLVGLDDFVAANNGKDMLIMLHQMGNHGPAYFKRYDEKFAKFTPVCEGNELAKCEHQSLINAYDNALLATDDFIAQSIQWLQTHSNAYDVSMLYVSDHGESLGENGVYLHGMPNAFAPKEQRSVPAFFWTDKQTGITPMATDTVLTHDAITPTLLKLFDVTADKVKDRTAFIR

>WP_140423330.1 MCR-1.21 human China

MMQHTSVWYRRSVSPFVLVASVAVFLTATANLTFFDKISQTYPIADNLGFVLTIAVVLFGAMLLITTLLSSYRYVLKPVLILLLIMGAVTSYFTDTYGTVYDTTMLQNALQTDQAETKDLLNAAFIMRIIGLGVLPSLLVAFVKVDYPTWGKGLMRRLGLIVASLALILLPVVAFSSHYASFFRVHKPLRSYVNPIMPIYSVGKLASIEYKKASAPKDTIYHAKDAVQATKPDMRKPRLVVFVVGETARADHVSFNGYERDTFPQLAKIDGVTNFSNVTSCGTSTAYSVPCMFSYLGADEYDVDTAKYQENVLDTLDRLGVSILWRDNNSDSKGVMDKLPKAQFADYKSATNNAICNTNPYNECRDVGMLVGLDDFVAANNGKDMLIMLHQMGNHGPAYFKRYDEKFAKFTSVCEGNELAKCEHQSLINAYDNALLATDDFIAQSIQWLQTHSNAYDVSMLYVSDHGESLGENGVYLHGMPNAFAPKEQRSVPAFFWTDKQTGITPMATDTVLTHDAITPTLLKLFDVTADKVKDRTAFIR

>MCS1110827.1 MCR-1 chicken Pakistan

MMQHTSVWYRRSVSPFVLVASVAVFLTATANLTFFDKISQTYPIADNLGFVLTIAVVLFGAMLLITTLLSSYRYVLKPVLILLLIMGAVTSYFTDTYGTVYDTTMLQNALQTDQAATKDLLNAAFIMRIIGLGVLPSLLVAFVKVDYPTWGKGLMRRLGLIVASLALILLPVVAFSSHYASFFRVHKPLRSYVNPIMPIYSVGKLASIEYKKASAPKDTIYHAKDAVQATKPDMRKPRLVVFVVGETARADHVSFNGYERDTFPQLAKIDGVTNFSNVTSCGTSTAYSVPCMFSYLGADEYDVDTAKYQENVLDTLDRLGVSILWRDNNSDSKGVMDKLPKAQFADYKSATNNAICNTNPYNECRDVGMLVGLDDFVAANNGKDMLIMLHQMGNHGPAYFKRYDEKFAKFTPVCEGNELAKCEHQSLINAYDNALLATDDFIAQSIQWLQTHSNAYDVSMLYVSDHGESLGENGVYLHGMPNAFAPKEQRSVPAFFWTDKQTGITPMATDTVLTHDAITPTLLKLFDVTADKVKDRTAFIR

>WDV57765.1 MCR-1 sus scrofa Thailand

MRQHTSVWYRRSVSPFVLVASVAVFLTATANLTFFDKISQTYPIADNLGFVLTIAVVLFGAMLLITTLLSSYRYVLKPVLILLLIMGAVTSYFTDTYGTVYDTTMLQNALQTDQAETKDLLNAAFIMRIIGLGVLPSLLVAFVKVDYPTWGKGLMRRLGLIVASLALILLPVVAFSSHYASFFRVHKPLRSYVNPIMPIYSVGKLASIEYKKASAPKDTIYHAKDAVQATKPDMRKPRLVVFVVGETARADHVSFNGYERDTFPQLAKIDGVTNFSNVTSCGTSTAYSVPCMFSYLGADEYDVDTAKYQENVLDTLDRLGVSILWRDNNSDSKGVMDKLPKAQFADYKSATNNAICNTNPYNECRDVGMLVGLDDFVAANNGKDMLIMLHQMGNHGPAYFKRYDEKFAKFTPVCEGNELAKCEHQSLINAYDNALLATDDFIAQSIQWLQTHSNAYDVSMLYVSDHGESLGENGVYLHGMPNAFAPKEQRSVPAFFWTDKQTGITPMATDTVLTHDAITPTLLKLFDVTADKVKDRTAFIR

>HAW5961415.1 MCR-1 human Poland

MMQHTSVWYRRSVSPFVLVASVAVFLTATANLTFFDKISQTYPIADNLGFVLTIAVVLFGAMLLITTLLSSYRYVLKPVLILLLIMGAVTSYFTDTYGTVYDTTMLQNALQTDQAETKDLLNAAFIMRIIGLGVLPSLLVAFVKVDYPTWGKGLMRRLGLIVASLALILLPVVAFSSHYASFFRVHKPLRSYVNPIMPIYSVGKLASIEYKKASAPKDTIYHAKDAVQATKPDMRKPRLGVFVVGETARADHVSFNGYERDTFPQLAKIDGVTNFSNVTSCGTSTAYSVPCMFSYLGADEYDVDTAKYQENVLDTLDRLGVSILWRDNNSDSKGVMDKLPKAQFADYKSATNNAICNTNPYNECRDVGMLVGLDDFVAANNGKDMLIMLHQMGNHGPAYFKRYDEKFAKFTPVCEGNELAKCEHQSLINAYDNALLATDDFIAQSIQWLQTHSNAYDVSMLYVSDHGESLGENGVYLHGMPNAFAPKEQRSVPAFFWTDKQTGITPMATDTVLTHDAITPTLLKLFDVTADKVKDRTAFIR

>MCS1115617.1 MCR-1 chicken Pakistan

MMQHTSVWYRRSVSPFVLVASVAVFLTATANLTFFDKISQTYPIADNLGFVLTIAVVLFGAMLLITTLLSSYRYVLKPVLILLLIMGAVTSYFTDTYGTVYDTTMLQNALQTVQAETKDLLNAAFIMRIIGLGVLPSLLVAFVKVDYPTWGKGLMRRLGLIVASLALILLPVVAFSSHYASFFRVHKPLRSYVNPIMPIYSVGKLASIEYKKASAPKDTIYHAKDAVQATKPDMRKPRLVVFVVGETARADHVSFNGYERDTFPQLAKIDGVTNFSNVTSCGTSTAYSVPCMFSYLGADEYDVDTAKYQENVLDTLDRLGVSILWRDNNSDSKGVMDKLPKAQFADYKSATNNAICNTNPYNECRDVGMLVGLDDFVAANNGKDMLIMLHQMGNHGPAYFKRYDEKFAKFTPVCEGNELAKCEHQSLINAYDNALLATDDFIAQSIQWLQTHSNAYDVSMLYVSDHGESLGENGVYLHGMPNAFAPKEQRSVPAFFWTDKQTGITPMATDTVLTHDAITPTLLKLFDVTADKVKDRTAFIR

>HAW5734332.1 MCR-1 human Poland

MMQHTSVWYRRSVSPFVLVASVAVFLTATANLTFFDKISQTYPIADNLGFVLTIAVVLFGAMLLITTLLSSYRYVLKPVLILLLIMGAVTSYFTDTYGTVYDTTMLQNALQTDQAETKDLLNAAFIMRIIGLGVLPSLLVAFVKVDYPTWGKGLMRRLGLIVASLALILLPVVAFSSHYASFFRVHKPLRSYVNPIMPIYSVCKLASIEYKKASAPKDTIYHAKDAVQATKPDMRKPRLVVFVVGETARADHVSFNGYERDTFPQLAKIDGVTNFSNVTSCGTSTAYSVPCMFSYLGADEYDVDTAKYQENVLDTLDRLGVSILWRDNNSDSKGVMDKLPKAQFADYKSATNNAICNTNPYNECRDVGMLVGLDDFVAANNGKDMLIMLHQMGNHGPAYFKRYDEKFAKFTPVCEGNELAKCEHQSLINAYDNALLATDDFIAQSIQWLQTHSNAYDVSMLYVSDHGESLGENGVYLHGMPNAFAPKEQRSVPAFFWTDKQTGITPMATDTVLTHDAITPTLLKLFDVTADKVKDRTAFIR

>HAZ3295069.1 MCR-1 food Spain

MMQHTSVWYRRSVSPFVLVASVAVFLTATANLTFFDKISQTYPIADNLGFVLTIAVVLFGAMLLITTLLSSYRYVLKPVLILLLIMGAVTSYFTDTYGTVYDTTMLQNALQTDQAETKDLLNAAFIMRIIGLGVLPSLLVAFVKVDYPTWGKGLMRRLGLIVASLALILLPVVAFSSHYASFFRVHKPLRSYVNPIMPIYSVGKLASIEYKKASAPKDTIYHAKDAVQATKPDMRKPRLVVFVVGETARADHVSFNGYERDTFPQLAKIDGVTNFSNVTSCGTSTAYSVPCMFSYLGADEYDVDTAKYQENVLDTLDRLGVSILWRDNNSDSKGVMDKLPKAQFADYKSATNNAICNTNPYNECRDVGMLVGLDDFVAANNGKDMLIMLHQMGNHGPAYFKRYDEKFAKFTPVCEGNELAKCEHQSLINAYDNALLATDDFIAQSIQWLQTHSNAYDVSMLYVSDHGESLGENGVYLHGMQNAFAPKEQRSVPAFFWTDKQTGITPMATDTVLTHDAITPTLLKLFDVTADKVKDRTAFIR

>HBN1924940.1 MCR-1 gallus Bangladesh

MMQHTSVWYRRSVSPFVLVASVAVFLTATANLTFFDKISQTYPIADNLGFVLTIAVVLFGAMLLITTLLSSYRYVLKPVLILLLIMGAVTSYFTDTYGTVYDTTMLQNALQTDQAETKDLLNAAFIMRIIGLGVLPSLLVAFVKVDYPTWGKGLMRRLGLIVASLALILLPVVAFSSHYASFFRVHKPLRSYVNPIMPICSVGKLASIEYKKASAPKDTIYHAKDAVQATKPDMRKPRLVVFVVGETARADHVSFNGYERDTFPQLAKIDGVTNFSNVTSCGTSTAYSVPCMFSYLGADEYDVDTAKYQENVLDTLDRLGVSILWRDNNSDSKGVMDKLPKAQFADYKSATNNAICNTNPYNECRDVGMLVGLDDFVAANNGKDMLIMLHQMGNHGPAYFKRYDEKFAKFTPVCEGNELAKCEHQSLINAYDNALLATDDFIAQSIQWLQTHSNAYDVSMLYVSDHGESLGENGVYLHGMPNAFAPKEQRSVPAFFWTDKQTGITPMATDTVLTHDAITPTLLKLFDVTADKVKDRTAFIR

>HBB9511317.1 MCR-1 human Poland

MMQHTSVWYRRSVSPFVLVASVAVFLTATANLTFFDKISQTYPIADNLGFVLTIAVVLFGAMLLITTLLSSYRYVLKPVLILLLIMGAVTSYFTDTYGTVYDTTMLQNALQTDQAETKDLLNAAFIMRIIGLGVLPSLLVAFVKVDYPTWGKGLMRRLGLIVASLALILLPVVAFSSHYASFFRVHKPLRSYVNPIMPIYSVGKLASIEYKKASAPKDTIYHAKDAVQATKPDMRKPRLVVFVVGETARADHVSFNGYERDTFPQLAKIDGVTNFSNVTSCGTSTAYSVPCMFSYLGADEYDVDTAKYQENVLDTLDRLGVSILWRDNNSDSKGVMDKLPKAQFADYKSATNNAICNTNPYNECRDVGMLVGLDDFVAANNGKDMLIMLHQMGNHGPAYFKRYDEKFAKFTPVCEGNELAKCEHQSLINAYDNALLATDDFIAQSIQWLQTHSNAYDVSMLYVSDHGESLGENGVYLHGMPNAFAPKEQRSVPACFWTDKQTGITPMATDTVLTHDAITPTLLKLFDVTADKVKDRTAFIR

>WP_106743337.1 MCR-1.18 chicken China

MMQHTSVWDRRSVSPFVLVASVAVFLTATANLTFFDKISQTYPIADNLGFVLTIAVVLFGAMLLITTLLSSYRYVLKPVLILLLIMGAVTSYFTDTYGTVYDTTMLQNALQTDQAETKDLLNAAFIMRIIGLGVLPSLLVAFVKVDYPTWGKGLMRRLGLIVASLALILLPVVAFSSHYASFFRVHKPLRSYVNPIMPIYSVGKLASIEYKKASAPKDTIYHAKDAVQATKPDMRKPRLVVFVVGETARADHVSFNGYERDTFPQLAKIDGVTNFSNVTSCGTSTAYSVPCMFSYLGADEYDVDTAKYQENVLDTLDRLGVSILWRDNNSDSKGVMDKLPKAQFADYKSATNNAICNTNPYNECRDVGMLVGLDDFVAANNGKDMLIMLHQMGNHGPAYFKRYDEKFAKFTPVCEGNELAKCEHQSLINAYDNALLATDDFIAQSIQWLQTHSNAYDVSMLYVSDHGESLGENGVYLHGMPNAFAPKEQRSVPAFFWTDKQTGITPMATDTVLTHDAITPTLLKLFDVTADKVKDRTAFIR

>ARX60875.1 mcr-1.5 human Argentina

MQHTSVWYRRSVSPFVLVASVAVFLTATANLTFFDKISQTYPIADNLGFVLTIAVVLFGAMLLITTLLSSYRYVLKPVLILLLIMGAVTSYFTDTYGTVYDTTMLQNALQTDQAETKDLLNAAFIMRIIGLGVLPSLLVAFVKVDYPTWGKGLMRRLGLIVASLALILLPVVAFSSHYASFFRVHKPLRSYVNPIMPIYSVGKLASIEYKKASAPKDTIYHAKDAVQATKPDMRKPRLVVFVVGETARADHVSFNGYERDTFPQLAKIDGVTNFSNVTSCGTSTAYSVPCMFSYLGADEYDVDTAKYQENVLDTLDRLGVSILWRDNNSDSKGVMDKLPKAQFADYKSATNNAICNTNPYNECRDVGMLVGLDDFVAANNGKDMLIMLHQMGNHGPAYFKRYDEKFAKFTPVCEGNELAKCEHQSLINAYDNALLATDDFIAQSIQWLQTYSNAYDVSMLYVSDHGESLGENGVYLHGMPNAFAPKEQRSVPAFFWTDKQTGITPMATDTVLTHDAITPTLLKLFDVTADKVKDRTAFIR

>HBB1918590.1 MCR-1 poultry China

MMQHTSVWYRRSVSPFVLVASVAVFLTATANLTFFDKISQTYPIADNLGFVLTIAVVLFGAMLLITTLLSSYRYVLKPVLILLLIMGAVTSYFTDTYGTVYDTTMLQNALQTDQAETKDLLNAAFIMRIIGLGVLPSLLVAFVKVDYPTLGKGLMRRLGLIVASLALILLPVVAFSSHYASFFRVHKPLRSYVNPIMPIYSVGKLASIEYKKASAPKDTIYHAKDAVQATKPDMRKPRLVVFVVGETARADHVSFNGYERDTFPQLAKIDGVTNFSNVTSCGTSTAYSVPCMFSYLGADEYDVDTAKYQENVLDTLDRLGVSILWRDNNSDSKGVMDKLPKAQFADYKSATNNAICNTNPYNECRDVGMLVGLDDFVAANNGKDMLIMLHQMGNHGPAYFKRYDEKFAKFTPVCEGNELAKCEHQSLINAYDNALLATDDFIAQSIQWLQTHSNAYDVSMLYVSDHGESLGENGVYLHGMPNAFAPKEQRSVPAFFWTDKQTGITPMATDTVLTHDAITPTLLKLFDVTADKVKDRTAFIR

>EFO5013314.1 MCR-1 gallus USA

MMQHTSVWYRRSVSPFVLVASVAVFLTATANLTFFDKISQTYPIADNLGFVLTIAVVLFGAMLLITTLLSSYRYVLKPVLILLLIMGAVTSYFTDTYGTVYDTTMLQNALQTDQAETKDLLNAAFIMRIIGLGVLPSLLVAFVKVDYPTWGKGLMRRLGLIVASLALILLPVVAFSSHYASFFRVHKPLRSYVNPIMPIYSVGKLASIEYKKASAPKDTIYHAKDAVQATKPDMRKPRLVVFVVGETARADHVSFNGYERDTFPQLAKIDGVTNFSNVTSCGTSTAYSVPCMFSYLGADEYDVDTAKYQENVLDTLDRLGVSILWRDNNSDSKGVMDKLPKAQFADYKSATNNAICNTNPYNECRDVGMLVGLDDFVAANNGKDMLIMLHQMGNHGPAYFKRYDEKFAKFTPVCEGNELAKCEHQSLINAYDNALLATDDFIAQSIQWLQTHSNAYDVSMLYVSDHGESLGENGVYLHGMPNAFAPQEQRSVPAFFWTDKQTDITPMATDTVLTHDAITPTLLKLFDVTADKVKDRTAFIR

>WP_099982815.1 MCR-1.11 human Peru

MMQHTSVVWYRRSVSPFVLVASVAVFLTATANLTFFDKISQTYPIADNLGFVLTIAVVLFGAMLLITTLLSSYRYVLKPVLILLLIMGAVTSYFTDTYGTVYDTTMLQNALQTDQAETKDLLNAAFIMRIIGLGVLPSLLVAFVKVDYPTWGKGLMRRLGLIVASLALILLPVVAFSSHYASFFRVHKPLRSYVNPIMPIYSVGKLASIEYKKASAPKDTIYHAKDAVQATKPDMRKPRLVVFVVGETARADHVSFNGYERDTFPQLAKIDGVTNFSNVTSCGTSTAYSVPCMFSYLGADEYDVDTAKYQENVLDTLDRLGVSILWRDNNSDSKGVMDKLPKAQFADYKSATNNAICNTNPYNECRDVGMLVGLDDFVAANNGKDMLIMLHQMGNHGPAYFKRYDEKFAKFTPVCEGNELAKCEHQSLINAYDNALLATDDFIAQSIQWLQTHSNAYDVSMLYVSDHGESLGENGVYLHGMPNAFAPKEQRSVPAFFWTDKQTGITPMATDTVLTHDAITPTLLKLFDVTADKVKDRTAFIR

>HCN7793754.1 MCR-1 gallus Ecuador

MMQHTSVWYRRSVSPFVLVASVAVFLTATANLTFFDKISQTYPIADNLGFVLTIAVVLFGAMLLITTLLSSYRYVLKPVLILLLIMGAVTSYFTDTYGTVYDTTMLQNALQTDQAETKDLLNAAFIMRIIGLGVLPSLLVAFVKVDYPTWGKGLMRRLGLIVASLALILLPVVAFSSPYASFFRVHKPLRSYVNPIMPIYSVGKLASIEYKKASAPKDTIYHAKDAVQATKPDMRKPRLVVFVVGETARADHVSFNGYERDTFPQLAKIDGVTNFSNVTSCGTSTAYSVPCMFSYLGADEYDVDTAKYQENVLDTLDRLGVSILWRDNNSDSKGVMDKLPKAQFADYKSATNNAICNTNPYNECRDVGMLVGLDDFVAANNGKDMLIMLHQMGNHGPAYFKRYDEKFAKFTPVCEGNELAKCEHQSLINAYDNALLATDDFIAQSIQWLQTHSNAYDVSMLYVSDHGESLGENGVYLHGMPNAFAPKEQRSVPAFFWTDKQTGITPMATDTVLTHDAITPTLLKLFDVTADKVKDRTAFIR

>HAW5632264.1 MCR-1 human Poland

MMQHTSVWYRRSVSPFVLVASVAVFLTATANLTFFDKISQTYPIADNLGFVLTIAVVLFGAMLLITTLLSSYRYVLKPVLILLLIMGAVTSYFTDTYGTVYDTTMLQNALQTDQAETKDLLNAAFIMRIIGLGVLPSLLVAFVKVDYPTWGKGLMRLLGLIVASLALILLPVVAFSSHYASFFRVHKPLRSYVNPIMPIYSVGKLASIEYKKASAPKDTIYHAKDAVQATKPDMRKPRLVVFVVGETARADHVSFNGYERDTFPQLAKIDGVTNFSNVTSCGTSTAYSVPCMFSYLGADEYDVDTAKYQENVLYTLDRLGVSILWRDNNSDSKGVMDKLPKAQFADYKSATNNAICNTNPYNECRDVGMLVGLDDFVAANNGKDMLIMLHQMGNHGPAYFKRYDEKFAKFTPVCEGNELAKCEHQSLINAYDNALLATDDFIAQSIQWLQTHSNAYDVSMLYVSDHGESLGENGVYLHGMPNAFAPKEQRSVPAFFWTDKQTGITPMATDTVLTHDAITPTLLKLFDVTADKVKDRTAFIR

>HBI2739951.1 MCR-1 human Netherlands

MMQHTSVWYRRSVSPFVLVASVAVFLTATANLTFFDKISQTYPIADNLGFVLTIAVVLFGAMLLITTLLSSYRYVLKPVLILLLIMGAVTSYFTDTYGTVYDTTMLQNALQTDQAETKDLLNAAFIMRIIGLGVLPSLLVAFVKVDYPTWGKGLMRRLGLIVASLALILLPVVAFSSHYASFFRVHKPLRSYVNPIMPIYSVGKLASIEYKKASAPKDTIYHAKDAVQATKPDMRKPRLVVFVVGETARADHVSFNGYERDTFPQLAKIDGVTNFSNVTSCGTSTAYSVPCMFSYLGADEYDVDTAKYQENVLDTLDRLGVSILWRDNNSYSKGVMDKLPKAQFADYKSATNNAICNTNPYNECRDVGMLVGLDDFVAANNGKDMLIMLHQMGNHGPAYFKRYDEKFAKFTPVCEGNELAKCEHQSLINAYDNALLATDDFIAQSIQWLQTHSNAYDVSMLYVSDHGESLCENGVYLHGMPNAFAPKEQRSVPAFFWTDKQTGITPMATDTVLTHDAITPTLLKLFDVTADKVKDRTAFIR

>HBB8281335.1 MCR-1 food China

TSVWYRRSVSPFVLVASVAVFLTATANLTFFDKISQTYPIADNLGFVLTIAVVLFGAMLLITTLLSSYRYVLKPVLILLLIMGAVTSYFTDTYGTVYDTTMLQNALQTDQAETKDLLNAAFIMRIIGLGVLPSLLVAFVKVDYPTWGKGLMRRLGLIVASLALILLPVVAFSSHYASFFRVHKPLRSYVNPIMPIYSVGKLASIEYKKASAPKDTIYHAKDAVQATKPDMRKPRLVVFVVGETARADHVSFNGYERDTFPQLAKIDGVTNFSNVTSCGTSTAYSVPCMFSYLGADEYDVDTAKYQENVLDTLDRLGVSILWRDNNSDSKGVMDKLPKAQFADYKSATNNAICNTNPYNECRDVGMLVGLDDFVAANNGKDMLIMLHQMGNHGPAYFKRYDEKFAKFTPVCEGNELAKCEHQSLINAYDNALLATDDFIAQSIQWLQTHSNAYDVSMLYVSDHGESLGENGVYLHGMPNAFAPKEQRSVPAFFWTDKQTGITPMATDTVLTHDAITPTLLKLFDVTADKVKDRTAFIR

>ATX68181.1 MCR-1 chicken Lebanon

QHTSVWYRRSVSPFVLVASVAVFLTATANLTFFDKISQTYPIADNLGFVLTIAVVLFGAMLLITTLLSSYRYVLKPVLILLLIMGAVTSYFTDTYGTVYDTTMLQNALQTDQAETKDLLNAAFIMRIIGLGVLPSLLVAFVKVDYPTWGKGLMRRLGLIVASLALILLPVVAFSSHYASFFRVHKPLRSYVNPIMPIYSVGKLASIEYKKASAPKDTIYHAKDAVQATKPDMRKPRLVVFVVGETARADHVSFNGYERDTFPQLAKIDGVTNFSNVTSCGTSTAYSVPCMFSYLGADEYDVDTAKYQENVLDTLDRLGVSILWRDNNSDSKGVMDKLPKAQFADYKSATNNAICNTNPYNECRDVGMLVGLDDFVAANNGKDMLIMLHQMGNHGPAYFKRYDEKFAKFTPVCEGNELAKCEHQSLINAYDNALLATDDFIAQSIQWLQTHSNAYDVSMLYVSDHGESLGENGVYLHGMPNAFAPKEQRSVPAFFWTDKQTGITPMATDTVLTHDAITPTLLKLFDVTADKVKDRT

>HBB4100187.1 MCR-1 pig Vietnam

VWYRRSVSPFVLVASVAVFLTATANLTFFDKISQTYPIADNLGFVLTIAVVLFGAMLLITTLLSSYRYVLKPVLILLLIMGAVTSYFTDTYGTVYDTTMLQNALQTDQAETKDLLNAAFIMRIIGLGVLPSLLVAFVKVDYPTWGKGLMRRLGLIVASLALILLPVVAFSSHYASFFRVHKPLRSYVNPIMPIYSVGKLASIEYKKASAPKDTIYHAKDAVQATKPDMRKPRLVVFVVGETARADHVSFNGYERDTFPQLAKIDGVTNFSNVTSCGTSTAYSVPCMFSYLGADEYDVDTAKYQENVLDTLDRLGVSILWRDNNSDSKGVMDKLPKAQFADYKSATNNAICNTNPYNECRDVGMLVGLDDFVAANNGKDMLIMLHQMGNHGPAYFKRYDEKFAKFTPVCEGNELAKCEHQSLINAYDNALLATDDFIAQSIQWLQTHSNAYDVSMLYVSDHGESLGENGVYLHGMPNAFAPKEQRSVPAFFWTDKQTGITPMATDTVLTHDAITPTLLKLFDVTADKVKDRTAFIR

>APC57542.1 MCR-1 chicken China

MWYRRSVSPFVLVASVAVFLTATANLTFFDKISQTYPIADNLGFVLTIAVVLFGAMLLITTLLSSYRYVLKPVLILLLIMGAVTSYFTDTYGTVYDTTMLQNALQTDQAETKDLLNAAFIMRIIGLGVLPSLLVAFVKVDYPTWGKGLMRRLGLIVASLALILLPVVAFSSHYASFFRVHKPLRSYVNPIMPIYSVGKLASIEYKKASAPKDTIYHAKDAVQATKPDMRKPRLVVFVVGETARADHVSFNGYERDTFPQLAKIDGVTNFSNVTSCGTSTAYSVPCMFSYLGADEYDVDTAKYQENVLDTLDRLGVSILWRDNNSDSKGVMDKLPKAQFADYKSATNNAICNTNPYNECRDVGMLVGLDDFVAANNGKDMLIMLHQMGNHGPAYFKRYDEKFAKFTPVCEGNELAKCEHQSLINAYDNALLATDDFIAQSIQWLQTHSNAYDVSMLYVSDHGESLGENGVYLHGMPNAFAPKEQRSVPAFFWTDKQTGITPMATDTVLTHDAITPTLLKLFDVTADKVKDRTAFIR

>AVR64822.1 MCR-1 human China

MWYRRSVSPFVLVASVAVFLTATANLTFFDKISQTYPIADNLGFVLTIAVVLFGAMLLITTLLSSYRYVLKPVLILLLIMGAVTSYFTDTYGTVYDTTMLQNALQTDQAETKDLLNAAFIMRIIGLGVLPSLLVAFVKVDYPTWGKGLMRRLGLIVASLALILLPVVAFSSHYASFFRVHKPLRSYVNPIMPIYSVGKLASIEYKKASAPKDTIYHAKDAVQATKPDMRKPRLVVFVVGETARADHVSFNGYERDTFPQLAKIDGVTNFSNVTSCGTSTAYSVPCMFSYLGADEYDVDTAKYQENVLDTLDRLGVSILWRDNNSDSKGVMDKLPKAQFANYKSATNNAICNTNPYNECRDVGMLVGLDDFVAANNGKDMLIMLHQMGNHGPAYFKRYDEKFAKFTPVCEGNELAKCEHQSLINAYDNALLATDDFIAQSIQWLQTHSNAYDVSMLYVSDHGESLGENGVYLHGMPNAFAPKEQRSVPAFFWTDKQTGITPMATDTVLTHDAITPTLLKLFDVTADKVKDRTAFIR

>WP_241224137.1 MCR-1 human China

RRSVSPFVLVASVAVFLTATANLTFFDKISQTYPIADNLGFVLTIAVVLFGAMLLITTLLSSYRYVLKPVLILLLIMGAVTSYFTDTYGTVYDTTMLQNALQTDQAETKDLLNAAFIMRIIGLGVLPSLLVAFVKVDYPTWGKGLMRRLGLIVASLALILLPVVAFSSHYASFFRVHKPLRSYVNPIMPIYSVGKLASIEYKKASAPKDTIYHAKDAVQATKPDMRKPRLVVFVVGETARADHVSFNGYERDTFPQLAKIDGVTNFSNVTSCGTSTAYSVPCMFSYLGADEYDVDTAKYQENVLDTLDRLGVSILWRDNNSDSKGVMDKLPKAQFADYKSATNNAICNTNPYNECRDVGMLVGLDDFVAANNGKDMLIMLHQMGNHGPAYFKRYDEKFAKFTPVCEGNELAKCEHQSLINAYDNALLATDDFIAQSIQWLQTHSNAYDVSMLYVSDHGESLGENGVYLHGMPNAFAPKEQRSVPAFFWTDKQTGITPMATDTVLTHDAITPTLLKLFDVTADKVKDRTAFIR
